# Genome Sequence of a Lancefield Group C *Streptococcus zooepidemicus* Strain Causing Epidemic Nephritis: New Information about an Old Disease

**DOI:** 10.1371/journal.pone.0003026

**Published:** 2008-08-21

**Authors:** Stephen B. Beres, Ricardo Sesso, Sergio Wyton L. Pinto, Nancy P. Hoe, Stephen F. Porcella, Frank R. DeLeo, James M. Musser

**Affiliations:** 1 Center for Molecular and Translational Human Infectious Diseases Research, The Methodist Hospital Research Institute and Department of Pathology, Houston, Texas, United States of America; 2 Division of Nephrology, Escola Paulista de Medicina, Universidade Federal de Sao Paulo, Sao Paulo, Brazil; 3 Division of Nephrology, Hospital Sao Joao de Deus, Divinopolis, Brazil; 4 Division of Occupational Health and Safety, Office of Research Services, National Institutes of Health, Hamilton, Montana, United States of America; 5 Laboratory of Human Bacterial Pathogenesis, Rocky Mountain Laboratories, National Institute of Allergy and Infectious Diseases, National Institutes of Health, Hamilton, Montana, United States of America; Centre for DNA Fingerprinting and Diagnostics, India

## Abstract

Outbreaks of disease attributable to human error or natural causes can provide unique opportunities to gain new information about host-pathogen interactions and new leads for pathogenesis research. Poststreptococcal glomerulonephritis (PSGN), a sequela of infection with pathogenic streptococci, is a common cause of preventable kidney disease worldwide. Although PSGN usually occurs after infection with group A streptococci, organisms of Lancefield group C and G also can be responsible. Despite decades of study, the molecular pathogenesis of PSGN is poorly understood. As a first step toward gaining new information about PSGN pathogenesis, we sequenced the genome of *Streptococcus equi* subsp. *zooepidemicus* strain MGCS10565, a group C organism that caused a very large and unusually severe epidemic of nephritis in Brazil. The genome is a circular chromosome of 2,024,171 bp. The genome shares extensive gene content, including many virulence factors, with genetically related group A streptococci, but unexpectedly lacks prophages. The genome contains many apparently foreign genes interspersed around the chromosome, consistent with the presence of a full array of genes required for natural competence. An inordinately large family of genes encodes secreted extracellular collagen-like proteins with multiple integrin-binding motifs. The absence of a gene related to *speB* rules out the long-held belief that streptococcal pyrogenic exotoxin B or antibodies reacting with it singularly cause PSGN. Many proteins previously implicated in GAS PSGN, such as streptokinase, are either highly divergent in strain MGCS10565 or are not more closely related between these species than to orthologs present in other streptococci that do not commonly cause PSGN. Our analysis provides a comparative genomics framework for renewed appraisal of molecular events underlying APSGN pathogenesis.

## Introduction

Epidemics of infectious disease attributable to human error or natural causes are unfortunate, however such outbreaks can provide unique opportunities to gain new information about host-pathogen interactions and accelerated leads for pathogenesis research. Between December 1997, and July 1998, 253 cases of acute nephritis were identified in Nova Serrana, a small rural Brazilian community [1]. Illness was severe, of 133 confirmed cases, 3 were fatal, 7 required dialysis, and 96 were hospitalized. Extensive investigation by Brazilian health officials and personnel deployed by the United States Centers for Disease Control and Prevention linked the nephritis epidemic to consumption of a locally produced cheese made with unpasteurized milk. Illness was attributed to contamination of the cheese with a strain of Lancefield group C *Streptococcus equi* subspecies *zooepidemicus* [2]. *S. equi* subspecies *zooepidemicus* is primarily an opportunistic pathogen of a wide variety of non-human animal species, including important domesticated species such as horses, cows, pigs, sheep, and dogs, and as such is a pathogen of veterinary concern. It is a well-known cause of mastitis in cows and mares, and is the most frequently isolated opportunistic pathogen of horses [3]. *S. zooepidemicus* is a rare cause of human invasive infections such as bacteremia and meningitis, usually originating from zoonotic transmission from domesticated animals to humans. There are only a few dozen reports of sporadic cases in the literature for the last 30 years [4]–[29]. Importantly organisms of this species have also caused epidemic outbreaks of infection commonly associated with consumption of un- or inadequately pasteurized milk or milk products, and many of these epidemic outbreaks have been complicated with poststreptococcal glomerulonephritis (PSGN) [30]–[38].

PSGN research has a rich history but the disease has long defied definitive pathogenesis explanation at the molecular level (reviewed in [39], [40]). Dating back for almost a hundred and fifty years (predating our ability to distinguish between some streptococcal species) physicians and epidemiologists have repeatedly noted an association between both sporadic and epidemic beta-hemolytic streptococcal infections and subsequent acute glomerulonephritis [41]–[51]. However the streptococcal bacterium has never been isolated from the kidney at any stage of PSGN renal disease. Although PSGN usually occurs after infection with group A streptococci, organisms of Lancefield group C and G also can be responsible. Unlike common pyelonephritis, PSGN is not a purulent infection and the causative bacterial agent is not present in the kidney at the site of renal damage. In most cases the initial streptococcal infection has been cleared by the time patients exhibit symptoms of nephritis. Patients with PSGN commonly have albuminuria, kidney failure, hypertension, and edema usually occurring a few-to-several weeks after streptococcal infection of the throat or skin. The disease is widely believed to be caused when antibody–antigen immune complexes become lodged in the kidney glomerulus, trigger proinflammatory immunologic processes, and produce organ injury (alternative immune mechanisms have also been proposed see [39], [40]). As a consequence of the presumed immune-mediated mechanism, extensive research conducted over decades has been directed toward identifying streptococcal antigens that elicit the inciting immunologic trigger. Toward this end, many extracellular streptococcal products have been causally implicated, including streptokinase, streptococcal pyrogenic exotoxin B (SpeB, an extracellular cysteine protease), glyceraldehyde-3-phosphate dehydrogenase (GAPDH), and others. However, none of these molecules has been shown unambiguously to be the cause of PSGN. Inasmuch as so little is known about the molecular pathogenesis of PSGN, the goal of the present study was to begin to accelerate research into this disease by sequencing the genome of a streptococcal strain responsible for a devastating and large nephritis outbreak.

## Results and Discussion

### Overview of the Genome of Strain MGCS10565

**Figure 1:**
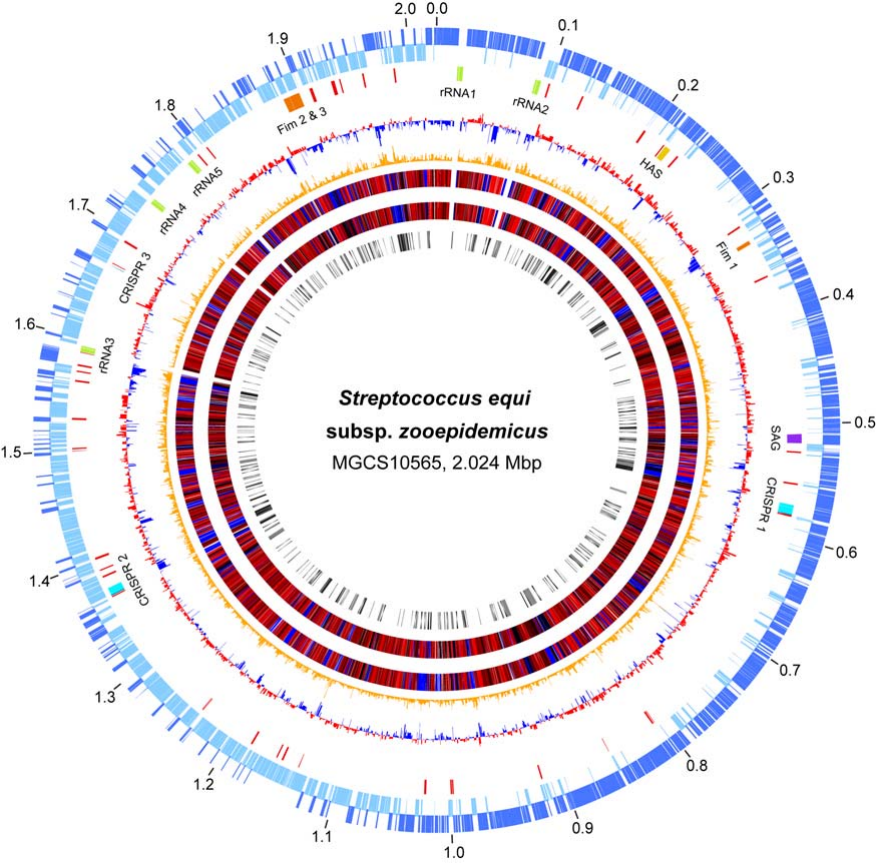
Genome atlas. Data from outermost-to-innermost circles are in the following order. Genome size in megabase pairs (circle 1). Annotated CDSs encoded on the forward (circle 2) and reverse (circle 3) chromosomal strands are in dark and light blue respectively. Reference landmarks (circle 4) as labeled are, ribsomal RNAs in green, fimbrial operons in orange, hyaluronic acid capsule synthesis loci in gold, CRISPR/CAS phage immunity loci in light blue, streptolysin S (*sag*) operon in purple, and ISs/transposons in red. CDS percent G+C content (circle 5) with greater and lesser than average in red and blue, respectively. Net divergence of CDS dinucleotide composition (circle 6) from the average is in orange. TBLASTN comparison of gene content with nephritogenic GAS serotype M12 strain MGAS2096 (circle 7), with high similarity in red and low in and blue. TBLASTN comparison of gene content with other sequenced streptococcal species (circle 8), with high similarity in red and low in blue. Species-specific gene content (circle 9), products not present in the other streptococcal species are in black and products sharing less than 50% amino acid identity with the most similar streptococcal homologue are in gray.

The genome of *Streptococcus equi* subspecies *zooepidemicus* strain MGCS10565 is a single, circular chromosome of 2,024,171 bp (Figure 1). The genome size is very close to the 2.00 Mbp average among the streptococcal strains for which complete genome sequences are publicly available (*n* = 31, Table 1). The G+C content of the genome is 42.59%, a value closely similar to the G+C content (∼41%) of the genome of two other equine strains of *Streptococcus equi* that are under investigation (www.sanger.ac.uk/Projects/S_zooepidemicus/ and www.sanger.ac.uk/Projects/S_equi/). In comparison to the major human pathogenic streptococcal species, the genome is modestly larger than genomes of group A *Streptococcus pyogenes* (GAS) (ave. 1.88 Mbp, *n* = 12 strains), and smaller than those of group B *Streptococcus agalactiae* (GBS) (ave. 2.17 Mbp, *n* = 3 strains) or *Streptococcus pneumoniae* (ave. 2.12 Mbp, *n* = 4 strains). The G+C content is at the high end for the genus, being ∼1-to-8% greater than that of all of the other streptococcal genomes except *Streptococcus sanguinis*. The genome has 1961 predicted protein coding sequences (CDSs) with an average gene length of 878 bp, coding for an average product of 292 amino acids (range, 37-to-1634 aa). In the aggregate coding sequence constitutes 85.0% of the genome. These coding sequence values are very similar to the averages for the genus (Table 1).

**Table 1:** Sequenced Streptococci.

Group	Species and Strain	Length (nt)	G+C %	Coding Sequence				rRNAs	tRNA	Acc. No.	Ref.
				% Genome	No.	Ave. (nt)	No./kb				
Pyogenic	*S. equi zooepidemicus* MGCS10565	2,024,171	42.59	85.0	1,961	878	0.97	5	57		—
Pyogenic	*S. equi equi* 4047	2,253,793	41.28	85.9	2,238	865	0.99	6	66	—	Sanger*
Pyogenic	*S. pyogenes* SF370	1,852,441	38.51	83.7	1,697	914	0.92	6	67	AE004092	[137]
Pyogenic	*S. pyogenes* MGAS5005	1,838,554	38.53	86.6	1,865	854	1.01	6	67	CP000017	[83]
Pyogenic	*S. pyogenes* MGAS10270	1,928,252	38.43	87.4	1,986	848	1.03	6	67	CP000260	[56]
Pyogenic	*S. pyogenes* MGAS315	1,900,521	38.59	85.8	1,865	874	0.98	6	67	AE014074	[138]
Pyogenic	*S. pyogenes* SSI-1	1,894,275	38.55	84.9	1,861	864	0.98	5	57	BA000034	[139]
Pyogenic	*S. pyogenes* MGAS10750	1,937,111	38.32	87.4	1,979	855	1.02	6	67	CP000262	[56]
Pyogenic	*S. pyogenes* Manfredo	1,841,271	38.63	83.7	1,745	883	0.95	6	67	AM295007	[140]
Pyogenic	*S. pyogenes* MGAS10394	1,899,877	38.69	87.1	1,886	878	0.99	6	67	CP000003	[117]
Pyogenic	*S. pyogenes* MGAS2096	1,860,355	38.73	87.3	1,898	856	1.02	6	67	CP000261	[56]
Pyogenic	*S. pyogenes* MGAS9429	1,836,467	38.54	87.7	1,877	858	1.02	6	67	CP000259	[56]
Pyogenic	*S. pyogenes* MGAS8232	1,895,017	38.55	85.2	1,845	875	0.97	6	67	AE009949	[141]
Pyogenic	*S. pyogenes* MGAS6180	1,897,573	38.35	86.9	1,894	871	1.00	6	67	CP000056	[118]
Pyogenic	*S. agalactiae* A909	2,127,839	35.62	86.2	1,995	918	0.94	7	80	CP000114	[57]
Pyogenic	*S. agalactiae* NEM316	2,211,485	35.63	87.7	2,094	926	0.95	7	80	AL732656	[77]
Pyogenic	*S. agalactiae* 2603 V/R	2,160,267	35.65	86.5	2,124	880	0.98	7	80	AE009948	[78]
Pyogenic	*S. uberis* 0140J	1,852,352	36.63	89.8	1,869	890	1.01	5	58	—	Sanger*
Mitis	*S. gordonii* Challis	2,196,662	40.51	88.0	2,051	942	0.93	4	59	CP000725	[64]
Mitis	*S. pneumoniae* D39	2,046,115	39.71	83.6	1,914	894	0.94	4	58	CP000410	[62]
Mitis	*S. pneumoniae* Hungary19-A6	2,245,615	39.63	82.8	2,155	863	0.96	4	58	CP000936	JCVI†
Mitis	*S. pneumoniae* R6	2,038,615	39.72	86.9	2,043	867	1.00	4	58	AE007317	[61]
Mitis	*S. pneumoniae* TIGR4	2,160,842	39.70	83.5	2,104	857	0.97	4	58	AE005672	[63]
Mitis	*S. sanguinis* SK36	2,388,435	43.40	88.7	2,270	933	0.95	4	61	CP000387	[65]
—	*S. suis* 05ZYH33	2,096,309	41.11	87.7	2,186	841	1.04	4	56	CP000407	[142]
—	*S. suis* 98HAH33	2,095,698	41.11	87.7	2,185	841	1.04	4	56	CP000408	[142]
—	*S. suis* P1/7	2,007,491	41.30	88.4	1,969	902	0.98	4	56	—	Sanger*
Mutans	*S. mutans* UA159	2,030,921	36.83	85.9	1,960	890	0.97	5	65	AE014133	[60]
Salivarius	*S. thermophilus* CNRZ1099	1,796,226	39.08	83.9	1,915	787	1.07	6	67	CP000024	[66]
Salivarius	*S. thermophilus* LMD9	1,856,368	39.08	76.9	1,710	835	0.92	6	67	CP000419	[67]
Salivarius	*S. thermophilus* LMG18311	1,796,846	39.09	84.0	1,889	799	1.05	6	67	CP000023	[66]

* www.sanger.ac.uk/Projects/S_equi; www.sanger.ac.uk/Projects/S_uberis; www.sanger.ac.uk/Projects/S_suis.

† Unpublished

The origin of replication (*oriC*) was inferred to be located in the intergenic region upstream of *dnaA* (Sez_0001) on the basis of GC skew and the clustering of seven DnaA box motifs flanking this gene. The start of nucleotide numbering was selected to correspond with the published GAS genomes. The exact nucleotide position of the terminus of replication (*terC*) is unknown. *terC* was inferred to lie upstream and adjacent to *murM* (Sez_1015) on the basis of shift in GC skew and a corresponding reversal in the predominant coding DNA strand. Given these replication initiation and termination assignments, the forward replicore (1,052 kbp) is slightly larger than the reverse (972 kbp). The orientation of genes is biased, with 78.7% (1546/1961) of the genes transcribed in the direction of DNA replication. This bias is stronger on the forward (825/1014, 81.4%) than the reverse replicore (721/947, 76.1%). The genome has five ribosomal RNA operons, compared to four in *S. pneumoniae*, six in GAS, and seven in GBS. All five rRNA operons are clustered in a ∼0.5 Mbp genome quadrant flanking *oriC*, a position that effectively increases the copy number of the rRNA operons during exponential growth. The genome encodes 57 tRNAs, which is similar to the 58 present in *S. pneumoniae*, but fewer than the 67 present in GAS and the 80 in GBS. These 57 tRNAs correspond to all of the 20 standard amino acids, but encompass only 31 of the 63 sense codons of the universal genetic code. Genes encoding tRNAs for cysteine, histidine, proline, and tryptophan are present in single copies. Most of the tRNAs, (78.9%) are located in close proximity to rRNAs (within 2 kbp of flanking sequence) which is also the case for GAS and GBS, but not *S. pneumoniae* (only ∼20%).

### Overview of Inferred Extracellular Proteins

Pathogenic streptococcal species produce an extensive array of extracellular proteins and use several protein export mechanisms. Many of these secreted, lipid-anchored, or cell-wall-anchored proteins are virulence factors. We identified 100 genes (comprising more than 5% of the genome) that would produce an inferred protein with an aminoterminal Sec-dependent secretion signal sequence (Table S1). These genes have an average G+C composition (42.9%) like that of the genome (42.6%), but encode proteins significantly longer in average length (478 amino acids versus 292 amino acids, respectively). Most of these proteins have predicted functions consistent with an extracellular location, and many have an exported ortholog in another streptococcal species. In addition, 44 of the 100 proteins have a canonical Gram-positive carboxyterminal sortase cell-wall-sorting signal with an LPxTG cleavage/anchoring motif. Among the streptococci, this is an exceptionally high number of putative cell wall anchored surface proteins. For example, it is 11 more than identified in *S. sanguinis*, two-to-three times the number identified in GAS, GBS, and *S. pneumoniae*, and more than seven times the number found in *S. mutans*. The genome encodes five sortases mediating cell-wall anchoring, including one A-family member (Sez_1020), and four C-family members (i.e. third sortase family HMM defined in [52]) (Sez_0813, Sez_1820, Sez_1826, and Sez_1827). Gram-positive genomes encode a single A-family sortase that catalyzes the wall anchoring of the majority of the cell surface proteins, whereas multiple C-family sortases can be encoded. In GBS and GAS C-family sortases are located in operons with fimbrial subunit proteins and are required for fimbrial synthesis. Consistent with this, all four of the C-family sortases present in MGCS10565 are encoded adjacent to genes encoding fimbrial subunit homologues (see below).

We identified 39 inferred proteins that have a lipoprotein aminoterminal secretion signal sequence with an LxAC cleavage/anchoring motif (Table S2). Most of these proteins also have inferred functions consistent with an extracellular location. For example, 21 of these 39 proteins are predicted substrate-binding components of ABC transporters used for importing sugars, amino acids/peptides, and metals. Streptococci and lactic acid bacteria commonly have secretion systems for peptides involved in bacteriocin production and quorum sensing. The peptides secreted by these systems have a double-glycine leader sequence that is proteolytically removed during secretion by dedicated ABC transporters with peptidase C39 domains [53]. Because of their small size and low similarity, genes encoding these types of peptides often are not annotated in genome sequences. Three genes encoding ABC transporters with a C39 peptidase domain were identified in the genome of strain MGCS10565. Fifteen genes encoding products of 43-to-98 amino acids with putative double-glycine leader peptides were identified either adjacent to these transporter genes or other putative competence/bacteriocin regulation, processing, or immunity genes (Table S3). This group of genes includes orthologues of pore-forming bacteriocins/cytolysins produced by other streptococci, most notably the key GAS virulence factor streptolysin S (SagA).

Specialized Sec-independent transport systems like that initially identified in *Mycobacterium tuberculosis* responsible for exporting ESAT-6-like virulence factors have been found in a variety of low G+C Gram-positive bacteria including *Bacillus anthracis*, *Staphylococcus aureus*, and *Streptococcus gordonii* [54]. ESAT-6/WXG100-family proteins generally are ∼100 amino acids long, have a centrally located WxG motif, and lack a secretion signal sequence. Gene clusters for ESAT-6-like systems often encode more than one ESAT-6/WXG100-family protein located proximal to a posited transport protein with 2-to-3 FstK/SpoIIIE-like domains. A cluster of 9 genes (Sez_0530-to-Sez_0538) with similarity to the EsxA/EsxB ESAT-6-like system of *S. aureus* is present in strain MGCS10565 [55]. This cluster has five genes that would produce leaderless proteins of ∼100 amino acids, and two genes (Sez_0530 and Sez_0537) that encode proteins with a central WxG motif. They flank a gene (Sez_0535) encoding a product of 1458 amino acids that has three FstK/SpoIIIE-like domains and shares 45% amino acid identity with EssC of *S. aureus* [55].

### Horizontal Gene Transfer, Mobile Genetic Elements, and Competence

Horizontal gene transfer (HGT) and recombination events have played a major role in the evolution of the pathogenic streptococci. Mobile genetic elements (MGEs) that promote HGT such as phages, integrative conjugative elements (ICEs), and insertions sequences, are prominent features of all of the sequenced streptococcal genomes. MGCS10565 has ∼67 CDSs at 45 loci throughout the genome encoding complete or partial IS elements including multiple copies of IS*861*, IS*1548*, IS*1239*, IS*1193D*, IS*1202*, IS*Sth1*, and IS*Mbov3* (Figure 1 circle 4, Table S4). This number of IS elements is about twice that commonly present in GAS and GBS genomes, but about half that of *S. pneumoniae* genomes. Most (∼70%) of the ISs in strain MGCS10565 have intact transposases. Many of the ISs are located in close proximity to genes predicted to produce cell surface proteins, suggesting acquisition of these genes by HGT.

Prophages and ICEs comprise ∼10% of the GAS and GBS genomes [56], [57]. These elements encode the majority of the variably present strain-specific gene content within these species. This variant gene content contributes to biomedically relevant differences in phenotypes such as virulence and antimicrobial resistance. Importantly and unexpectedly, the genome of strain MGCS10565 lacks discernable phages or ICEs analogous to those identified in GAS and GBS (∼30-to-60-kbp regions encoding proteins characteristic of these MGEs, usually flanked by integration site short direct sequence repeats). Although genes homologous to phage integrase, replication, or repressor genes are present at more than a dozen loci throughout the genome, many are not full-length and none occur in a cluster of more than three contiguous phage genes. This result is somewhat surprising given that the MGCS10565 genome has three genes encoding proteins that are most similar to GAS phage-encoded secreted virulence factors (two DNases and a phospholipase A_2_, Sez_ 0668, Sez_0755, and Sez_1876, respectively), and phages are prevalent in many strains of the very closely related *S. equi* subsp. *equi* [58], [59]. However, this finding is consistent with the results of recent investigations that detected homologues of one or more GAS phage-encoded superantigens in most isolates of *S. equi* studied, but rarely in *S. zooepidemicus* [58]. Thus, the lack of prophages in the MGCS10565 genome may be the common condition for *S. zooepidemicus* strains.

Prophages are not present in any of the seven sequenced genomes of the naturally transformable streptococci, including *S. pneumoniae*, *S. gordonii*, *S. sanguinis*, or *S. mutans* [60]–[65]. In contrast, prophages are present in many of the sequenced genomes of streptococci for which natural transformation has not been demonstrated such as *S. thermophilus*, GAS, and GBS [56], [57], [66], [67]. This observation has led to the idea that systems exist in naturally transformable streptococci that provide resistance to uptake and incorporation of foreign DNA and may co-incidentally prevent stable prophage integration. The lack of prophages and the presence of many homologues of bacteriocin/competence genes in the MGCS10565 genome led us to hypothesize that *S. zooepidemicus* might be naturally competent. Consistent with this hypothesis, we identified homologues of 60 bacteriocin/competence genes present in clusters arrayed throughout the genome.

Successfully predicting the capacity for natural transformation based on gene content alone is problematic due to species-to-species variation in competence systems [68]. Among the streptococci, competence development is best characterized in *S. pneumoniae* where 23 genes have been determined by insertional mutagenesis and whole-genome microarray transcriptional analysis to be individually essential for transformation [69]. We identified one or more homologues of 22 of these 23 genes (Table 2). A homologue of *comW* was not present, however this does not preclude natural transformation as this gene is not required for *S. mutans* transformation. Several of the 60 putative competence associated proteins have upstream ComE or ComX consensus binding sites consistent with the possibility of a competence regulatory network (including the homologues of the ComE and ComG locus, CoiA, etc.).

**Table 2:** Essential Competence Genes.

	Genes	Product and/or Function	Spn TIGR4		% ID*	% SIM*	Sez MGCS10565	
			Locus Tag	Size aa			Locus Tag	Size aa
EARLY	comA	ABC transporter ATP-binding protein	SP_0042	717	81.5	91.8	Sez_0521	717
					65.9	82.6	Sez_1523	718
	comB	ABC transporter protein	SP_0043	449	49.0	69.9	Sez_0522	455
					31.2	53.0	Sez_1524	466
	comC	Competence stimulating pheromone	SP_2237	41	28.9	40.0	Sez_1525	44
					17.9	30.4	Sez_0518	46
					14.1	32.8	Sez_0520	55
	comD	TCS sensor histidine kinase	SP_2236	441	20.9	42.5	Sez_1526	444
					18.4	32.4	Sez_0582	370
	comE	TCS DNA-binding response regulator	SP_2237	250	33.9	55.6	Sez_1527	249
					16.4	31.1	Sez_0583	198
	comX1	Alternative sigma factor	SP_0014	159	35.8	59.3	Sez_1691	159
	comX2	Alternative sigma factor	SP_2006	159	35.8	59.3	Sez_1733	159
	comW	ComX activation/stabilization	SP_0018	67	–	–	–	–
LATE	comEA	Donor DNA-binding	SP_0954	216	39.0	59.3	Sez_0651	225
	comEC	Uptake permease/channel protein	SP_0955	746	46.4	64.7	Sez_0652	747
	comFA	ATP-binding DNA helicase/translocase	SP_2208	432	51.6	69.3	Sez_1547	440
	comFC	Competence protein	SP_2207	220	38.9	56.1	Sez_1546	193
	comGA/cglA	Traffic NTPase	SP_2053	313	60.4	76.0	Sez_0115	312
	comGB/cglB	Polytopic membrane protein	SP_2052	290	42.9	64.0	Sez_0116	328
	comGC/cglC	Major pseudopilin	SP_2051	108	57.4	75.0	Sez_0117	107
	comGD/cglD	Minor pseudopilin	SP_2050	134	39.0	57.4	Sez_0118	141
	comGG/cglG	Minor pseudopilin	SP_2047	137	29.7	49.3	Sez_0120	120
	coiA	Donor DNA processing	SP_0978	317	42.9	61.3	Sez_0660	325
	dprA	Donor DNA processing	SP_1266	286	57.5	75.6	Sez_0976	281
	cclA/pilD	Type IV prepilin peptidase	SP_1808	219	31.4	47.2	Sez_0584	213
	ssb	Single strand DNA-binding	SP_1908	131	71.0	82.4	Sez_1844	131
	recA	DNA recombinase	SP_1940	388	83.8	91.0	Sez_1873	378

* Determined using Needleman-Wunch global alignment.

Two different competence regulatory networks that use different but paralogous two-component systems (TCSs) are present in streptococci [70]. Competence induction in members of the mitis group, *S. pneumoniae*, *S. gordonii*, and *S. sanguinis* uses the ComD-ComE TCS sensor-regulator, whereas *S. mutans* uses a TCS that is more closely related to the *S. pneumoniae* bacteriocin-like peptide regulator BlpR-BlpH. Similarly, it has been suggested from indirect evidence that GAS strains containing the five-gene Blp-like streptococcal invasion locus (*silABCDE*), may also be naturally transformable [71]. We identified 31 genes comprising at least 14 TCS regulators in MGCS10565 (Table S5). Two to these TCS have characteristics that suggest a role in competence. The TCS encoded by Sez_1526-1527 although similar to *S. pneumoniae* ComDE is even more closely related to *S. pneumoniae* BlpRH. Sez_1527-to-Sez_1523 are homologous to *silABCDE* of GAS. This 6-kbp region has a 4% lower G+C composition than the genome average and is flanked on both sides by ISs, characteristics suggesting possible acquisition by HGT. Additionally present is a second TCS, Sez_0582-0583, that also might fill the role of ComDE. Sez_0582-0583 lack homologues sufficiently closely related in the NCBI NR database to permit prediction of function. A role for this TCS in competence is suggested on the basis of being flanked upstream by a putative peptide ABC transporter (Sez_0580-0581) and downstream by (Sez_0584) a gene encoding prepilin peptidase, an essential late product for formation of the competence pseudopilin DNA uptake apparatus. Thus, although speculative, the similarity to other naturally competent streptococci in the absence of prophage and the presence of genes essential for natural tranformation, argues for *S. zooepidemicus* being naturally competent.

Despite lacking prophages and ICEs, a considerable portion of the gene content of the MGCS10565 genome has characteristics suggesting it was acquired by HGT. For example, more than one-eighth of the gene content (272 CDSs) lacks a significant homologue in any sequenced genome of the other streptococcal species (strains in Table 1 except *S. equi subsp. equi*, TBLASTN comparison using a cutoff of *e* = 10^−9^). An additional 192 of the inferred products share less than 50% sequence identity with a product of another streptococcal species. This unique and divergent gene content (464 CDSs total) is distributed uniformly throughout the genome (Figure 1 circle 9). As a group it has a significantly atypical nucleotide composition (*P*<0.0001) relative to the genome and the gene content (1497 CDSs) that is more highly conserved with the genus (Figure S1). On average the *S. equi*-specific CDSs encode products that are 93 amino acids smaller and 3% lower in G+C composition than the genus-conserved CDSs. Approximately half of the *equi*-specific gene content encodes hypothetical proteins of unknown function. Genes encoding extracellular products are also very abundant in this group. Inferred extracellular proteins comprise a three-fold greater proportion of the unique and divergent CDSs than of the genus conserved CDSs (16.4% vs 5.3%). This differential is even more pronounced for the cell-wall-anchored set of proteins. Only 20% of these inferred cell surface proteins (9 of 44) have a homologue that shares greater than 50% global amino acid identity in another streptococcal species. Many of these proteins have a mosaic structure, being composed of domains conserved in other streptococcal surface proteins interspersed with unique and divergent domains.

### CRISPR Elements

Included in the MGCS10565 genome unique and divergent gene content are systems that likely provide resistance to the uptake and incorporation of foreign DNA. Clustered regular interspaced short palindromic repeat (CRISPR) elements and CRISPR-associated (CAS) genes constitute newly recognized and widely distributed prokaryotic systems that mediate resistance to infection by foreign DNA [72]. For example, CRISPR/CAS systems provide targeted phage immunity in *S. thermophilus* [73], [74]. The mechanism of resistance is poorly characterized but is posited to involve processes analogous to eukaryotic RNA interference. CRISPR/CAS systems are present in the genome sequences of *S. mutans*, *S. gordonii*, *S. thermophilus*, GBS, and GAS. We identified three CRISPR elements in MGCS10565, designated I (nt 581380–582010), II (nt 1366035–1367186), and III (nt 1669119–1669283) (Figure 1 circle 4). CRISPRs I and II are flanked by CAS genes (I = Sez_0548-to-Sez_0542, II = Sez_1327-to-Sez_1330). CRISPR/CAS elements are likely to be MGEs on the basis of phylogenetic analysis of CAS genes. It is noteworthy that CRISPR/CAS systems I and II are flanked, directly adjacent on one side and in close proximity on the other, by ISs suggesting they may be components of composite transposons. Further supporting the possibility of HGT, the identical 35-bp direct repeat of MGCS10565 CRISPR III also is present in some GAS strains. In the aggregate, the three CRISPR elements have 28 spacers, 4 of these spacers are nearly identical to sequences present in prophages predominantly of GAS and one matches phage CF32 of *S. equi* subsp. *equi*. A fifth spacer has 20 nts identical to a sequence in the SCC*mec*_N1_ element encoded by a *S. aureus* clone associated with the epidemic spread of methicillin resistant infections among injection drug users. Thus, our findings strongly suggest that *S. zooepidemicus* experiences infective exposure to phages related to those of GAS, consistent with the presence of GAS phage-encoded DNase and phospholipase A_2_ homologues in the MGCS10565 genome (described above).

### Phylogenetic Relationships of Strain MGCS10565 to Other Streptococci

**Figure 2:**
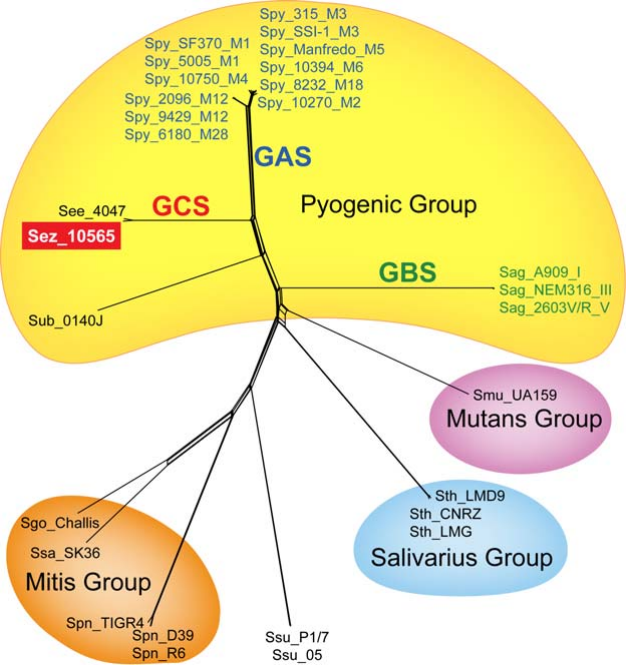
Streptococcal multilocus genetic relationships. Inferred sequences of the DNA replication and repair proteins DnaA, DnaE, DnaG, DnaI, DnaJ, DnaN, and DnaX were concatenated, aligned and used to infer genetic relationships among the streptococcal strains for which complete genome sequences are available. The genes encoding these proteins are conserved genus-wide and arrayed around the chromosome.

As an initial assessment of relatedness to other bacteria, we compared the inferred translated products of strain MGCS10565 with the combined NCBI nonredundant protein database using BLASTP. Approximately 11% (220 of 1961) of the products lacked a significant homologue in the database (defined as a cognate sharing greater than 25% global amino acid identity). Genes encoding these unique proteins are distributed throughout the genome. Most are hypothetical proteins of unknown function and 65% (142/220) are less than 100 amino acids in length. In contrast, 79% (1550/1961) of the MGCS10565 inferred proteins were significantly similar to the protein of another bacteria (defined as a cognate sharing greater than 50% global amino acid identity). The vast majority of the most similar proteins, ∼95% (1471/1550) were streptococcal homologues. Less than 3% of the products were most similar to an entry in the database from *S. equi* (subsp. *equi* or *zooepidemicus*), indicative of the paucity of genetic information previously available for this species. The percentages of most similar products in other streptococcal species descended in the following order: GAS 54.4%, GBS 5.9%, *S. suis* 3.1%, *S. mutans* 2.5%, *S. pneumoniae* 2.4%, and *S. thermophilus* 2.3%. All other streptococcal species combined accounted for less than 4% of the most similar homologues. We next performed a multilocus assessment of genetic relationships among the sequenced streptococci comparing the products of DNA replication and repair genes, *dnaA*, *dnaE*, *dnaG*, *dnaI*, *dnaJ*, *dnaK*, *dnaN*, and *dnaX* (Figure 2). These genes are arrayed around the genome and conserved across the genus. This showed that among the sequenced streptococci *S. zooepidemicus* is closely related to GAS *S. pyogenes* and GBS *S. agalactiae*. This is consistent with previous single locus estimates of streptococcal genetic affiliations based on comparisons of *sodA*, *rnpB*, or 16S rRNA sequences [75], [76].

**Figure 3:**
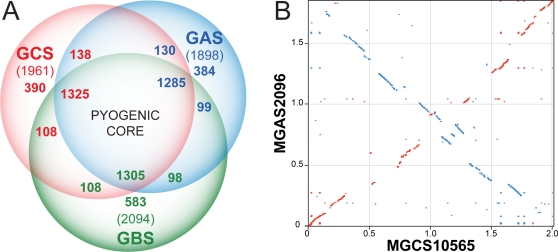
Genome comparisons. (A) Gene content comparison. The inferred proteomes of *S. zooepidemicus* (GCS), GAS, and *S. agalactiae* (GBS) were compared pair wise to the translated genomes of each other using TBLASTN (cutoff, *e* = 10^−9^). The numbers of genes given for each section are color coded to match the respective genomes. The numbers for CDS shared in common in the intersections differ slightly due to variance in gene copies species-to-species, such as resulting from gene duplication and mobile genetic element transfer events. (B) Aligned GCS and GAS genomes. The nucleotide sequence of the MGCS10565 (GCS) and MGAS2096 (GAS) genomes were compared and regions sharing at least 60% identity over a window of 30 nucleotides are illustrated. Conserved regions oriented in the same direction are in red, and regions opposite in direction are in blue.

We next compared the streptococcal pyogenic group *S. zooepidemicus*, GAS, and GBS genomes pair wise to assess commonality of gene content (Figure 3a). For this comparison, we used the genome of GAS strain MGAS2096, a nephritogenic strain of serotype M12 (a.k.a. Rockefeller strain A374) [56], and GBS strain NEM316 [77]. Sequences aligning with 75% of strain MGCS10565 inferred proteins are present in strain MGAS2096, and vice-versa. Similarly, sequences aligning with of 73% of strain MGCS10565 proteins are present in GBS strain NEM316. Although the number of putative common genes (∼1,430) is similar, the MGCS10565 proteins average ∼5% greater conservation with strain MGAS2096 than with strain NEM316 (59% identical and 67% similar versus 53% identical and 63% similar, respectively). We estimate that roughly one-fourth of the gene content of any one of these strains lacks orthologous content in the other two (range = 19.9-to-27.8%). Conversely about two-thirds of the gene content is shared. This common gene content constitutes a conserved pyogenic streptococcal core genome of about 1,300 genes, which is ∼300 more genes than *S. pneumoniae* was determined to have in common with GAS and GBS [78]. Specific orthologues of *S. zooepidemicus* genes present in GAS and vice-versa were identified as reciprocal-best-hit pairs in BLAST comparisons of the MGCS10565 and MGAS2096 genomes. On the basis of this criteria two-thirds of the MGCS10565 genes (1290 CDSs) have a MGAS2096 ortholog. Moreover 80% of these orthologous genes are closely flanked (within 3 CDSs) on both the 5′ and 3′ side by the same orthologs, thus slightly more than half of the MGCS10565 genes are conserved in local synteny with strain MGAS2096. Ninety percent of the orthologs (1172 CDSs) share greater than 50% global amino acid identity. Nearly all (94%) of the 182 genes encoded by prophages and ICEs in the MGAS2096 genome lack an ortholog in MGCS10565. On average, the orthologous gene products of these genomes share 74.9% amino acid identity. By comparison, reciprocal-best-hit pairs of GAS strains of different serotypes (i.e. GAS-to-GAS comparison) account for ∼80-to-85% of the genes, and on average share >95% global amino acid identity.

To determine the chromosomal location of conserved content, the *S. zooepidemicus* genome was aligned with itself, GAS strain MGAS2096, and *S. agalactiae* strain NEM316. The MGCS10565 self-alignment lacked large duplications or extensively repeated sequences such as the RUP or BOX elements found in *S. pneumoniae* genomes (not shown). The five rRNA operons were the largest repeated regions (5 kbp) identified. Alignment of the *S. zooepidemicus* and GAS genomes shows conserved chromosomal architecture (Figure 3b). The alignment has a classic X-pattern showing conserved regions located equidistant from *oriC*, consistent with the likelihood that these genomes have undergone multiple symmetric chromosomal inversions since they last shared a common ancestor [79]. Similar features were observed in the alignment with the *S. agalactiae* genome but conserved regions were reduced in size and symmetry (not shown).

### Proven and Putative Virulence Factors

Compared to the other pathogenic streptococci, relatively little is known about *S. zooepidemicus* factors mediating virulence in general and outbreaks of glomerulonephritis in particular. The MGCS10565 genome encodes over 100 genes homologous to putative and proven virulence factors of other pathogenic bacteria (Table S6). Consistent with participating in host-pathogen interactions, a preponderance of the genes (*n* = 70) encode products predicted to be extracellular. These genes encode factors likely to participate at several steps in pathogen-host interaction, including resistance to the host innate and adaptive immune responses (e.g. enzymes that degrade IgG, IL-8, C3, and C5a), adhesion (e.g. proteins with albumin, laminin, collagen, and fibronectin binding domains), host-cell toxicity (e.g. porins and hemolysins), invasion (e.g. internalin-like proteins), and dissemination (e.g. lipases, glycosidases, proteases, and nucleases). As a group these virulence gene homologues have a G+C composition virtually identical to the genome average (42.8% versus 42.6%), evidence against the idea that these genes were recently acquired by HGT from a genetically highly divergent donor organism. This also argues against very recent acquisition from the related major human pathogenic streptococci, GAS, GBS, and *S. pneumoniae* as they average 3-to-7% lower in G+C composition than *S. zooepidemicus*.

We found that nearly half of the identified putative virulence factors (*n* = 47) have a reciprocal-best-hit in the genome of GAS serotype M12 nephritogenic strain MGAS2096 (Table S6). Included are entire orthologous operons such as *hasABC* for synthesis of the antiphagocytic hyaluronic acid capsule and *sagABCDEFGHI* encoding the pore forming toxin streptolysin S, and individual genes like *spyCEP* and *scpA* encoding IL-8 protease and C5a peptidase, respectively. In the aggregate, these *S. zooepidemicus* products have only 64.0% global amino acid identity with their GAS orthologues, a value significantly less than the 74.9% identity of the average orthologous products of these strains. This level of divergence also argues against the likelihood that genes encoding these virulence factors were very recently transferred between GAS and strain MGCS10565.

**Figure 4:**
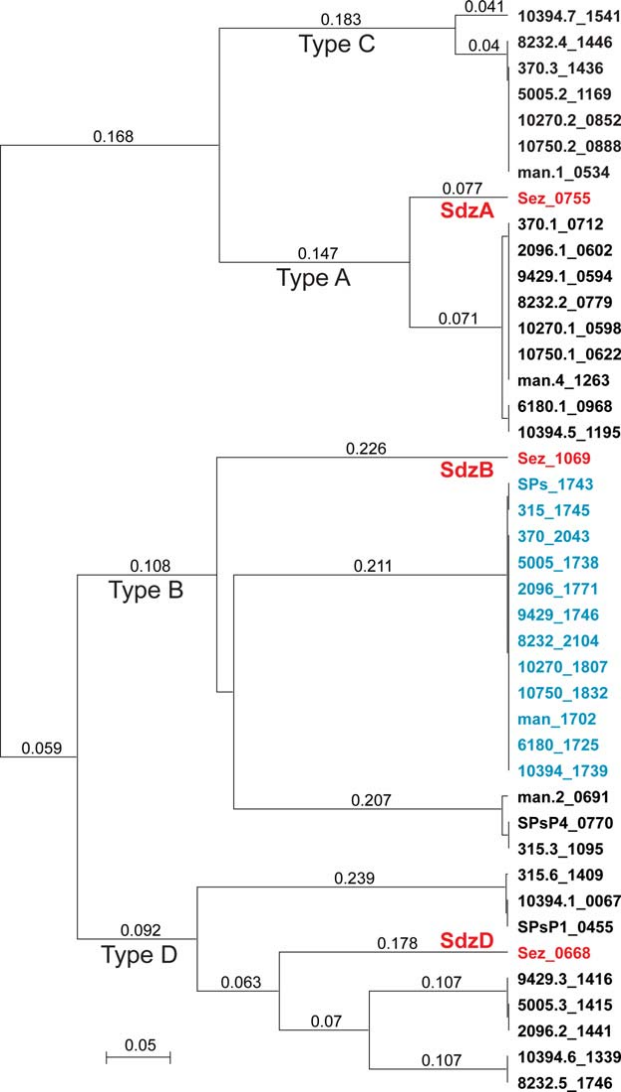
DNases genetic relationships. Inferred products of all of the DNase genes present in the genomes of the 12 sequenced GAS strains, chromosomally encoded (shown in blue) and prophage encoded (shown in black) were aligned with the those present in the *S. zooepidemicus* MGCS10565 genome (shown in red) and genetic relationships were inferred using the unweighted pair group method with arithmetic mean (UPGMA). Each of the *S. zooepidemicus* DNases is an outlier relative to the GAS DNases of the same type, arguing for an independent evolutionary path and against very recent horizontal transfer between the species.

Prophages are a primary mediator of GAS intra- and inter-serotype differences in virulence factor content [80]. Recently several GAS prophage-encoded virulence factors have been found in group C and G streptococci, including *S. equi* subspecies *equi and zooepidemicus* [58], [59], [81], [82]. In some instances these GCS virulence factors were phage-encoded and nearly identical to their GAS homologues suggesting very recent phage-mediated horizontal transfer. Importantly, these events may have contributed to the evolution of new virulent clones. Given that the *S. zooepidemicus* genome lacks prophage, but encodes two DNases and a phospholipase A_2_ that are most like GAS phage-encoded virulence factors, we examined these genes for evidence of recent phage mediated horizontal transfer. Secreted DNases made by GAS contribute to evasion of the host innate immune response in part by degrading neutrophil extracellular traps composed of DNA and antimicrobial histones [83]. Four secreted DNase types, designated A to D, have been described in GAS, and each of the sequenced GAS genome has multiple secreted DNases encoded on the chromosome and prophages. Strain MGCS10565 encodes three inferred secreted DNases each with a different reciprocal-best-hit ortholog in strain MGAS2096. Two of the three DNases (SdzD/Sez_0668 and SdzA/Sez_0755) have characteristics suggesting that they were horizontally transferred by phage. For example, their GAS orthologs are prophage-encoded, and moreover an alanyl-tRNA synthetase gene flanks Sez_0668 and a tRNA-Arg gene flanks Sez_0755. Genes encoding tRNAs and tRNA-synthetases are common targets of lambda-phage-like site-specific integrases. Lastly a paratox gene, so named because it is found in many GAS phages directly adjacent to secreted virulence toxins, also flanks Sez_0755. Inferred genetic relationships between these three DNases and those present in the GAS genomes are shown in Figure 4. Each DNase in strain MGCS10565 clearly clusters with a distinct GAS DNase type, consistent with descent from a common type-ancestor, but each *S. zooepidemicus* DNase branches separately from the GAS members of the type-cluster. These findings support the possibility of phage-mediated horizontal transfer, but the level of sequence divergence present in all three *S. zooepidemicus* DNases relative to their GAS orthologs argues against recent acquisition.

### Three Fimbriae-Encoding Regions

**Figure 5:**
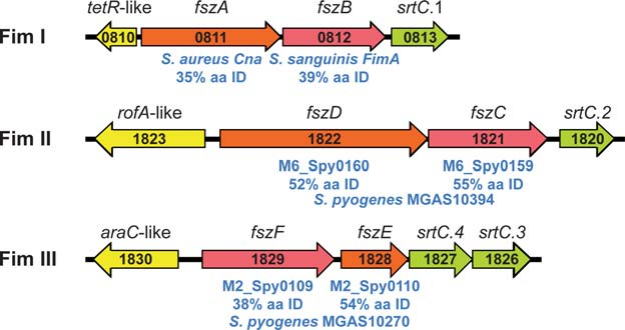
Schematic of three fimbrial operons. Illustrated are the 3 putative fimbrial operons identified in the MGCS10565 genome. Each operon has a gene encoding a protein homologous to the fimbrial backbone/major subunit protein (red) and an ancillary/minor subunit protein (orange). The genes encoding the fimbrial structural proteins are flanked at the 5′ end (but oriented in the opposite direction) by genes encoding regulatory proteins (yellow) and at the 3′ end by genes encoding sortases of the C-family (green). Gene numbers are given in black. Homologues of the structural proteins and their percent amino acid identity are given in blue. The putative major subunit proteins all have homology to GAS T-antigens, as expected.

Recent studies have revealed that several Gram-positive pathogens produce long extracellular structures resembling fimbriae that are composed of multiple protein subunits. These proteins mediate adhesion to components of the human extracellular matrix such as collagen and fibronectin and have become important targets of pathogenesis and vaccine research. The sequenced genomes of the fimbriae-producing strains of these species each have one or more operons encoding fimbrial structural proteins (a major subunit and one or more minor subunits), associated C-family sortases, and an adjacent upstream divergently-oriented regulator (often of the AraC/MsmR or RofA/Nra families) (reviewed in [84]). The structural proteins all have an aminoterminal secretion signal and a carboxyterminal sorting signal, and their assembly into fimbriae is dependent on the adjacently encoded dedicated sortases. The MGCS10565 genome has three operons with these characteristics suggesting the capacity to produce three distinct fimbriae (Figure 5). Putative Fim II and III operons have structural proteins related to fimbrial subunit proteins of the sequenced GAS serotype M6 and M2 genomes (∼50% amino acid identity and 70% amino acid similarity, respectively). The putative Fim I structural proteins do not have closely related GAS homologues. The Fim I minor subunit protein FszA (Cne) has similarity to *S. aureus* collagen-binding protein Cna (35% amino acid identity), and the major subunit protein FszB to *S. sanguinis* fimbrial protein FimA (40% amino acid identity). Production of two distinct fimbriae has been demonstrated for *Actinomyces naeslundii* [85] and *Corynebacterium diphtheriae* [86]. Moreover, *C. diphtheriae* strain NCTC13129 also is speculated to produce three distinct fimbriae. Thus, although production of genes for multiple distinct fimbriae has not been described in other streptococcal species, it has precedence among Gram-positive pathogens.

### A Remarkably Large Family of Extracellular Collagen-Like Proteins

Genes encoding collagen are ubiquitous in multicellular animals but rare in prokaryotes. At least 30 different collagen genes have been identified in the human genome. In contrast, a recent survey of 137 eubacterial genomes identified only 53 proteins with contiguous Gly-Xxx-Yyy collagen structural motif repeats (i.e. 53 prokaryotic collagen-like proteins, CLPs) [87], and very few bacterial genomes have multiple genes encoding CLPs. GAS has two cell-surface CLPs, SclA and SclB (Scl1 and Scl2), that form collagen-like triple helices. Various binding activities have been attributed to these two proteins, including in vitro interaction with α_2_β_1_ integrins of human fibroblasts and epithelial cells [88], [89]. This interaction triggers intracellular phosphorylation signaling cascades like those induced by integrin-binding of human ECM components.

**Figure 6:**
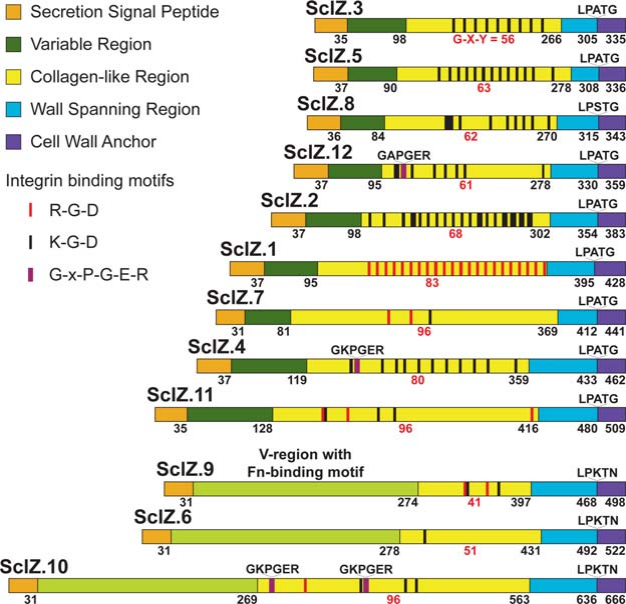
Schematic of collagen-like proteins. Illustrated are 12 inferred proteins with collagen structural motifs encoded by the MGCS10565 genome. These proteins are composed of the following domains (from amino- to carboxy-terminus): SSP, secretion signal peptide; V, variable region; CL, collagen-like region, W, a proline-rich putative cell wall spanning region, and finally a tripartite cell wall anchor. Numbers in black below the schematics are the last amino acid residue of each the respective regions. The number of contiguous Gly-Xxx-Yyy repeats composing the CL-regions are given in red. Sites within the CL-regions matching integrin recognition sequences RGD and KGD are indicated by red and black bars, respectively. Prokaryotic analogs (GxPGER) of human collagen sequences mediating high-affinity integrin binding, are indicated by violet bars. CLPs 6, 9, and 10 have V-regions with fibronectin-binding domains.

We identified 12 genes distributed throughout the MGCS10565 genome that encode inferred CLPs (Figure 6). These proteins, designated SclZ.1-to SclZ.12, each have a collagen-like region that varies in number of Gly-Xxx-Yyy repeats, primary sequence, and overall amino acid composition. The amino-terminal secretion signal, and carboxy-terminal cell wall anchoring regions of these proteins have significant similarity. Three of the 12 CLPs (SclZ.6, SclZ.9, and SclZ.10) share 30-to-60% pair wise amino acid identity across the region constituting the variable (V) region of the other CLPs. The V-region of these three proteins are larger than those of the other nine CLPs and each has similarity with a domain found in the amino-terminus of some streptococcal Fn-binding proteins (pfam08341). These are the first streptococcal CLPs to be identified that have a V-region matching a domain of known function. The variable regions of the other 9 CLPs lack significant similarity to other proteins in the NCBI NR database. With the exception of SclZ.6, all of the CLPs have multiple short amino acid sequences (RGD, KGD, and GxPGER) that mediate interactions with integrins (Figure 6). Remarkably, on average these CLPs have 10 integrin-binding sequence motifs (total = 118, range = 1-to-22). The motifs are located exclusively in the CL-regions of the proteins. Importantly, this is not a general feature of streptococcal CLPs, as no RGD or GxPGER sequences, and only four KGD sequences are present in the CL-regions of SclA (*n* = 3) and SclB (*n* = 1) of strain MGAS2096. Additionally only seven sites (RGD = 1 and KGD = 6) in total were found in two sets each composed of 12 proteins randomly selected from the MGCS10565 genome to match the size of the 12 CLPs (data not shown). Although the significance of these observations is not known, the large number strongly suggests an important role in host-pathogen interaction. Consistent with this it has recently been reported that human collagen bound to the GAS cell surface interacts with α2β1 integrins on endothelial cells inducing the uptake and transcytosis of GAS cells across endothelium [90]. Antibodies against the GAS and *S. equi* CLPs have been found in sera of infected humans and horses, respectively, indicating that these proteins are made in vivo during infection [91]. It is worth noting that antibodies against type IV collagen, an abundant component of the glomerular basement membrane, have been found in the sera of PSGN patients [92].

### Streptokinase

**Figure 7:**
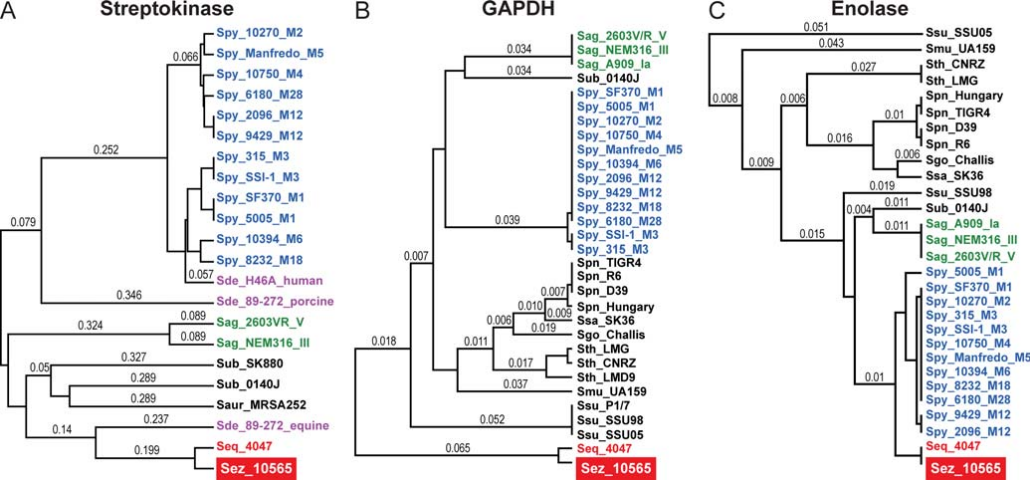
Genetic relationships of virulence factors implicated in PSGN pathogenesis. (A) Streptokinase, (B) GAPDH, and (C) Enolase. Inferred products for each of the virulence factors implicated in PSGN pathogenesis were aligned and relationships were inferred using the UPGMA method. GCS *S. equi* subsp. *equi* and *zooepidemicus* strains are shown in red, GAS *S. pyogenes* in blue, GBS *S. agalactiae* in green, GCS/GGS *S. dysgalactiae* subsp. *equisimilis* in purple, and other streptococcal species in black. These virulence factors are not more closely related between *S. zooepidemicus* and *S. pyogenes* than among the other streptococcal species.

Streptokinase has been implicated in PSGN pathogenesis by several investigators [39], [40]. SKN is produced by many beta-hemolytic group A, C, and G streptococci. SKN forms a 1:1 stoichometric complex with either plasminogen or plasmin, and in a host species-specific manner activates the conversion of plasminogen to plasmin a serine protease that can degrade fibrin clots and the extracellular matrix. SKN was implicated in nephritis as NSAP (however there is some confusion in the literature and nephritis-strain-associated-protein has also been suggested to be SpeB), a GAS extracellular protein initially thought to be uniquely produced by PSGN isolates [93]–[95]. In subsequent investigations it was shown that SKN is not uniquely produced by nephritogenic GAS strains, and moreover that sera from cases of APSGN, ARF, or healthy controls did not differ significantly in anti-SKN antibody titer [96]. The SKN gene is highly polymorphic and allelic variants display functional differences [97]. Current proponents of an SKN mediated PSGN mechanism posit that only certain variants of SKN are preferentially associated with nephritogenic strains of GAS [98], [99], but this is also equivocal [100], [101]. Consistent with host species-specific adaptation, SKN of strain MGCS10565 is most closely related to SKN found in *S. equi* subsp. *equi* and *S. dysgalactiae* subsp. *equisimilis* equine isolates, 90% and 51% amino acid identity respectively (Figure 7a). It bears little overall identity or similarity to SKN variants described in GAS or *S. dysgalactiae* subsp. *equisimilis* human or pig isolates, less than 15% amino acid identity. The lack of a close relationship of the *S. zooepidemicus* SKN variant with SKNs implicated in episodes of PSGN caused by other streptococcal strains such as GAS argues against a causal pathogenesis role of SKN.

### GAPDH/NAPlr

Highly varied findings have been reported concerning the association of GAS glyceraldehyde phosphate dehydrogenase (GAPDH, a.k.a. nephritis associated plasmin(ogen) receptor/NAPlr, preabsorbing antigen/PA-Ag) with PSGN. Multiple investigators have reported localizing GAPDH to glomeruli in renal biopsies of PSGN patients and elevated anti-GAPDH antibody titers in PSGN convalescent sera relative to normal healthy controls [102]–[105]. However other investigators using similar techniques specifically comparing SpeB and GAPDH within the same APSGN patient samples found an association with SpeB, but failed to detect an association with GAPDH [106]. The genome of *S. zooepidemicus* MGCS10565 encodes a GAPDH that shares 85.6% amino acid identity with the GAPDH made by strain GAS MGAS2096, a level of identity greater than average (74.9%) for orthologs of these two genomes (Figure 7b). However a modestly higher level of identity (86-to-88%) is shared between GAPDH of *S. zooepidemicus* and GBS, *S. pneumoniae*, *S. mutans*, or *S. gordonii* (in increasing order). Moreover GAPDH is virtually identical (>99.6%) among all twelve of the sequenced GAS genomes and in a comparison of GAPDH between GAS strains isolated from both APSGN and non-APSGN patients no obvious differences in expression were found [107]. Thus neither the level of GAPDH relatedness nor expression correlate well with streptococcal species that commonly cause APSGN versus those that do not, nor with nephritogenic versus non-nephritogenic serotypes of GAS.

### Enolase

Virtually all human cells express the glycolytic enzyme α-enolase, with kidney and thymus being the highest producers. In human tissues, α-enolase is present in the cytoplasm, and it is also expressed on cell surfaces where it acts as a plasminogen receptor. Antibodies against enolase are present in a wide variety of human autoimmune disorders (e.g. autoantibody-mediated nephritis) and infectious diseases [108], [109]. Enolase also occurs as a cytoplasmic and a cell surface associated protein in many bacteria including GAS, GBS, *S. pneumoniae* and *S. aureus*. The GAS cell-surface enolase (SEN) is a major plasminogen-binding protein that has 46% amino acid identity with human α-enolase [110]. Antibodies against GAS SEN cross-react with human α-enolase on cell surfaces, and elevated levels of antibodies against enolase were found in the sera of acute rheumatic fever patients relative to patients with GAS pharyngitis or healthy controls [111]. Together, these observations lead to the hypothesis that antibodies generated against GAS SEN may contribute to poststreptococcal autoimmune sequelae [111]. The *S. zooepidemicus* MGCS10565 genome encodes an enolase that has 98.6% amino acid identity with the SEN of GAS MGAS2096 (Figure 7c). This identity is far greater than the 74.9% average identity of orthologous proteins between these genomes. However a similar high level of identity, 96.8% and 94.3%, is shared with enolases of GBS and *S. pneumoniae*, respectively. Moreover SEN is virtually identical (>99%) among all twelve of the sequenced GAS genomes. Thus, although the MGCS10565 enolase is highly similar to that of MGAS2096, as with GAPDH, enolase relatedness does not correlate well with streptococcal species that commonly causes PSGN versus those that do not, nor with nephritogenic versus non-nephritogenic serotypes of GAS.

### Lack of a *speB* Gene

**Figure 8:**
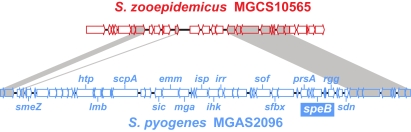
Schematic of the SpeB encoding region. Illustrated is an alignment of the SpeB encoding region of the *S. pyogenes* neprhitogenic serotype M12 strain MGAS2096 genome (shown in blue) with the corresponding region of the *S. zooepidemicus* strain MGCS10565 genome (shown in red). *speB* and several flanking genes (e.g. *smeZ*, *sof*, and *sfbX*) are not present in the *S. zooepidemicus* strain MGCS10565 genome.

SpeB also known as nephritis plasmin-binding protein (NPBP), is an extracellular cysteine protease that has been implicated as a pathogenic (causative) antigen in PSGN [39], [40]. NPBP was detected in the extracellular products of nephritis associated but not nonnephritogenic GAS [112]. Antibodies against SpeB are present in higher titer in the sera of APSGN patients relative to other infections and healthy controls [113]. This protein has also been detected in the glomeruli of patients with PSGN [112]. In addition, it has been claimed that SpeB is present in the culture supernatants of group C streptococci causing PSGN [114]. We made the very unexpected discovery that no *speB* gene or *speB* gene homologue was present in the genome of strain MGCS10565. Moreover, the genome of strain MGCS10565 lacks nearly all of a 46-kb chromosomal region corresponding to the area where *speB* and flanking genes are located in all GAS genomes characterized [115], [116]. The deleted region includes *speB*, *sof*, *sfbX*, and the entire Mga regulon (Figure 8), although as noted above orthologs of many of the virulence genes present in the Mga regulon (*mga*, *emm*, *scpA*, and *lmb*) are present elsewhere in the MGCS10565 genome (see Table S6). The lack of *speB* in strain MGCS10565 rules out the possibility that SpeB and/or anti-SpeB antibodies contributed to PSGN pathogenesis in these Brazilian epidemic patients. The results also call into question data suggesting that anti-SpeB antibodies contribute to PSGN pathogenesis in GAS-induced PSGN.

### Summary

In this work we describe the sequence of the genome of *S. equi* subsp. *zooepidemicus* strain MGCS10565, a Lancefield group C organism that caused a large epidemic of nephritis in Brazil. We discovered that the genome shares extensive gene content with genetically related GAS strains. However, strain MGCS10565 lacks prophages. We found that the genome has a large family of genes encoding secreted extracellular collagen-like proteins with multiple integrin-binding motifs. Importantly, the organism lacks a gene related to *speB*, thereby ruling out the prevailing idea that SpeB or antibodies reacting with it singularly cause PSGN. This comparative genome analysis provides a key genetic framework for reassessing our understanding of the molecular events contributing to PSGN pathogenesis.

## Materials and Methods

### Bacterial Strain

Strain MGCS10565 is a Lancefield group C *Streptococcus equi* subspecies *zooepidemicus* isolate from the throat of a patient with nephritis who was diagnosed during an epidemic of this disease in the state of Minas Gerais, Brazil. The strain is also designated CDC 5058 and has the same pulsed-field gel electrophoretic type and other genetic characteristics as organisms causing this outbreak [1], [2]. The strain has been deposited with the American Type Culture Collection and assigned catalog number ATCC BAA-1716.

### Genome Sequencing

We sequenced the genome of strain MGCS10565 to closure and an average Q40 value (a less than 1 in 10,000 predicted base call error rate) throughout by methods described previously [117], [118]. The genome was tiled by PCR after closure to validate the assembly. The genome sequence of strain MGCS10565 has been deposited in the GenBank database (accession no. CP001129).

### Genome Annotation and Bioinformatics Analysis

tRNAs, tmRNAs, and other noncoding RNAs were predicted using a combination of tRNAscan, ARAGORN, and INFERNAL comparison to Rfam models [119]–[121]. Ribosomal 5S, 16S, and 23S RNAs were predicted on the basis of similarity to GAS rRNAs using BLAST [122]. Coding sequences were predicted using Glimmer in conjunction with ELPH for selection of optimal start sites [123]. Predicted genes and intergenic regions were compared to the NCBI sequence database using blastcl3 and predicted CDS and start sites were adjusted accordingly. Secretion signal peptides were predicted using SignalP ([124] / www.cbs.dtu.dk/ services/SignalP/). Lipidation signal peptides were predicted using LipoP ([125] / www.cbs.dtu.dk/services/LipoP/). Sortases and cell wall sorting signals (i.e. sortase subtrates) were predicted with available hidden Markov models using HMMER ([52], [126] / bamics3.cmbi.kun.nl/jos/sortase_substrates/ help.html). Predicted transposase/insertion sequence annotations were based on comparisons to the ISfinder database (www-is.biotoul.fr). CRISPR elements were identified and CRISPR-associated gene annotations were based on comparisons made using CRISPRFinder ([127] / crispr.u-psud.fr/). Additional sequence predictions and comparison were obtained using two automated annotation systems, BaSYS ([128] / www.basys.com) and RAST ([129] / rast.nmpdr.org). Resultant bioinformatic predictions and automated annotations were integrated and curated using Artemis [130].

### Gene and Gene Content Comparisons

The stand-alone-BLAST set of applications (ftp.ncbi.nih.gov/blast/) was used to make streptococcal genome gene content comparisons. Results of various BLAST comparisons were parsed using BioParser ([131] / www.dbbm.fiocruz.br/BioParserWeb). Homologous gene content between genomes was identified using tblastn to allow for potential genome-to-genome annotation differences. Orthologous gene content was identified using blastp to identify reciprocal-best-hits in pair wise inferred proteome comparisons. The Euler-Venn applet was used to generate area proportional Venn diagrams (www.cs.kent.ac.uk/people/staff/pjr/EulerVennCircles/EulerVennApplet.html). CDS codon usage and nucleotide composition analyses were performed using CodonW (bioweb.pastuer.fr). The circular genome atlas was generated using GenomeViz [132]. Pairwise global protein alignments were performed using the “water” (Smith-Waterman) application of the EMBOSS bioinformatic suite [133]. Multiple sequence alignments were made using ClustalW [134], and phylogenetic reconstructions were made using SplitsTree [135]. Various additional comparisons and illustrations (e.g. dot matrix plots, CLP and fimbrial operon gene diagrams, etc…) were made using MacVector [136].

## Supporting Information

Figure S1 Species-specific and genus-conserved gene content comparison. (A) CDS length. (B) CDS percent G+C composition. (C) CDS dinucleotide composition; graphed is the net absolute difference from the average dinucleotide frequency summed for all 16 dinucleotide pairs at all three codon positions (the higher the value the more atypical the nucleotide composition). (D) CDS codon adaptation index. Abbreviations: U&D, unique and divergent products (n = 464) relative to other sequenced streptococcal species; Con, conserved products (n = 1497) relative to other sequenced streptococcal species; all products (n = 1961) of the MGCS10565 genome. Bars show the ranges of values, boxed horizontal lines show the means, and the boxes show the standard errors about the means. Accompanying tables give results of unpaired t-test with Welch's correction.
(0.40 MB PDF)

Table S1 Products with Predicted Sec-Dependent Secretion Signal Sequence. Inferred proteins with canonical amino-terminal Sec-dependent secretion signal sequence
(0.10 MB PDF)

Table S2 Products with Predicted Lipidation Signal Sequence. Inferred lipoproteins with with canonical amino-terminal lipidation signal sequence
(0.05 MB PDF)

Table S3 Products with Predicted Double-Glycine Secretion Signal Sequence. Inferred proteins with bacteriocin/competenece peptide-like double-glycine secretion signal sequence
(0.04 MB PDF)

Table S4 Insertion Sequence Elements
(0.09 MB PDF)

Table S5 Two component system regulator genes
(0.09 MB DOC)

Table S6 Proven and putative virulence factor homologues
(0.22 MB DOC)
