# Supplementary material for: Genome Sequence of a Lancefield Group C Streptococcus zooepidemicus Strain Causing Epidemic Nephritis: New Information about an Old Disease
Source: PLoS One. 2008 Aug 21;3(8):e3026. doi: 10.1371/journal.pone.0003026 (PMC2516327; doi:10.1371/journal.pone.0003026)
Supplement: Figure S1 — Species-specific and genus-conserved gene content comparison. (A) CDS length. (B) CDS percent G+C composition. (C) CDS dinucleotide composition; graphed is the net absolute difference from the average dinucleotide frequency summed for all 16 dinucleotide pairs at all three codon positions (the higher the value the more atypical the nucleotide composition). (D) CDS codon adaptation index. Abbreviations: U&D, unique and divergent products (n = 464) relative to other sequenced streptococcal species; Con, conserved products (n = 1497) relative to other sequenced streptococcal species; all products (n = 1961) of the MGCS10565 genome. Bars show the ranges of values, boxed horizontal lines show the means, and the boxes show the standard errors about the means. Accompanying tables give results of unpaired t-test with Welch's correction. (0.40 MB PDF) [file pone.0003026.s001.pdf]

A

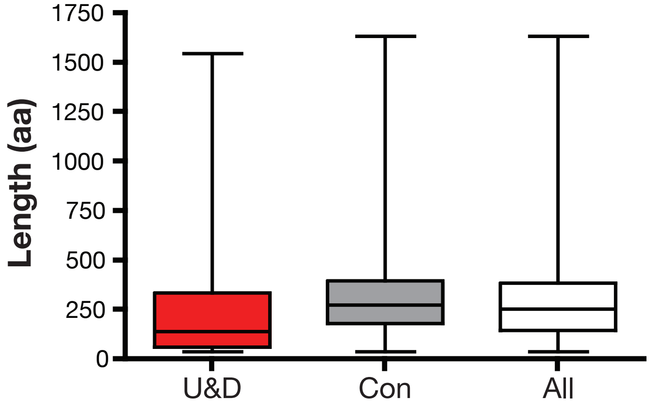

Group	Mean	Difference in Mean	P Value
All	291.7 ± 4.669	70.78 ± 11.21	< 0.0001
U&D	220.9 ± 10.19		
All	291.7 ± 4.669	21.94 ± 6.920	< 0.0001
Con	313.6 ± 5.108		
U&D	220.9 ± 10.19	92.71 ± 11.40	< 0.0001
Con	313.6 ± 5.108		

B

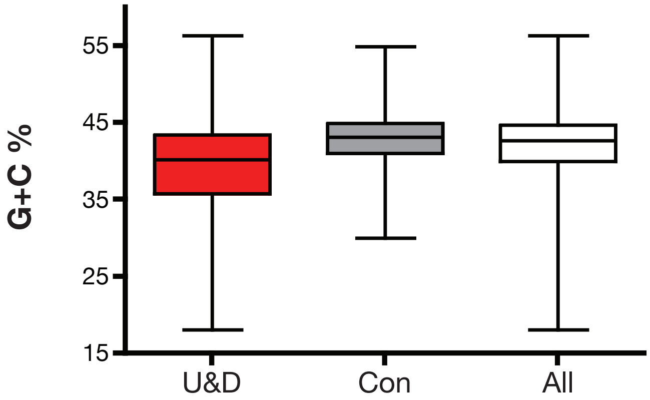

Group	Mean	Difference in Mean	P Value
All	42.03 ± 0.0928	2.397 ± 0.276	< 0.0001
U&D	39.63 ± 0.2600		
All	42.03 ± 0.0928	0.743 ± 0.1239	< 0.0001
Con	42.77 ± 0.0821		
U&D	39.63 ± 0.2600	3.139 ± 0.2726	< 0.0001
Con	42.77 ± 0.0821		

C

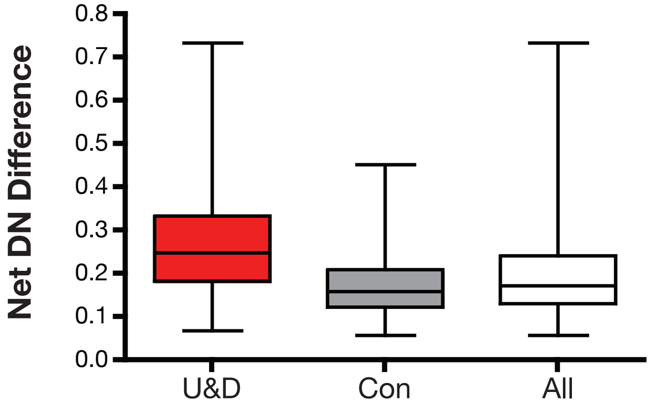

Group	Mean	Difference in Mean	P Value
All	0.1942 ± 0.0002	0.0729 ± 0.0054	< 0.0001
U&D	0.2671 ± 0.0050		
All	0.1942 ± 0.0002	0.0226 ± 0.0026	< 0.0001
Con	0.1716 ± 0.0017		
U&D	0.2671 ± 0.0050	0.0956 ± 0.0053	< 0.0001
Con	0.1716 ± 0.0017		

D

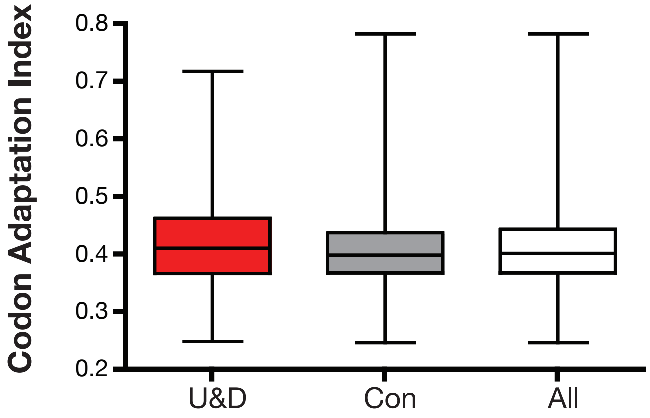

Group	Mean	Difference in Mean	P Value
All	0.4139 ± 0.0016	0.0055 ± 0.0038	< 0.1411
U&D	0.4195 ± 0.0034		
All	0.4139 ± 0.0016	0.0017 ± 0.0024	< 0.4844
Con	0.4122 ± 0.0018		
U&D	0.4195 ± 0.0034	0.0072 ± 0.0039	< 0.0606
Con	0.4122 ± 0.0018		
