# Supplementary material for: Genome Sequence of a Lancefield Group C Streptococcus zooepidemicus Strain Causing Epidemic Nephritis: New Information about an Old Disease
Source: PLoS One. 2008 Aug 21;3(8):e3026. doi: 10.1371/journal.pone.0003026 (PMC2516327; doi:10.1371/journal.pone.0003026)
Supplement: Table S1 — Products with Predicted Sec-Dependent Secretion Signal Sequence. Inferred proteins with canonical amino-terminal Sec-dependent secretion signal sequence (0.10 MB PDF) [file pone.0003026.s002.pdf]

Table S1. Products with Predicted Sec-Dependent Secretion Signal Sequence

Gene Tag	Length	Cleavage Site	CWA	Product/Function
Sez_0018	392aa	24-25: VGA-DD	--	muramidase
Sez_0029	384aa	38-39: VHA-AV	--	muramidase
Sez_0099	428aa	37-38: VHA-EA	LPATG	SclZ.1, streptococcal collagen-like protein of zooepidemicus 1
Sez_0100	383aa	37-38: VKA-DV	LPATG	SclZ.2, streptococcal collagen-like protein of zooepidemicus 2
Sez_0110	184aa	26-27: ANA-DA	--	hypothetical protein function unknown
Sez_0156	580aa	26-27: AQA-DV	--	PG endopeptidase/protease
Sez_0162	335aa	35-36: VQA-QE	LPATG	SclZ.3, streptococcal collagen-like protein of zooepidemicus 3
Sez_0164	392aa	37-38: VKA-DV	LPSTG	IgG-binding protein
Sez_0185	559aa	38-39: VGA-ET	LPATA	IgG-binding protein
Sez_0190	462aa	37-38: VKA-ED	LPATG	SclZ.4, streptococcal collagen-like protein of zooepidemicus 4
Sez_0213	336aa	37-38: AKA-EE	LPATG	SclZ.5, streptococcal collagen-like protein of zooepidemicus 5
Sez_0265	834aa	36-37: VKA-DD	--	surface exclusion protein
Sez_0310	147aa	44-45: VSA-ST	--	conserved protein function unknown
Sez_0313	576aa	37-38: VMA-DK	LPQTS	fibronenctin-binding protein
Sez_0338	326aa	41-42: VRA-DV	LPSTG	M-like protein
Sez_0370	471aa	27-28: VLA-ED	--	muramidase
Sez_0371	1251aa	26-27: VRS-EA	--	Fe(3+) siderophore
Sez_0372	293aa	29-30: AYA-DK	NPLSV	heme-binding protein
Sez_0432	98aa	29-30: KAA-NE	--	hypothetical protein function unknown
Sez_0446	345aa	32-33: VQA-ST	--	esterase/lipase
Sez_0475	250aa	37-38: PAA-IT	--	conserved protein possible protease

Gene Tag	Length	Cleavage Site	CWA	Product/Function
Sez_0499	482aa	31-32: ILA-EQ	LPRTN	Fnz-like, fibronectin-binding protein, factor H-binding protein
Sez_0500	522aa	31-32: VLA-EQ	LPKTN	SclZ.6, streptococcal collagen-like protein of zooepidemicus 6
Sez_0511	1634aa	34-35: VAA-EE	LPSTG	interleukin 8 protease
Sez_0513	394aa	33-34: VSA-AT	LPSTG	albumin-binding protein
Sez_0532	174aa	24-25: VLA-ND	--	conserved protein function unknown
Sez_0560	370aa	30-31: GYA-TS	--	secreted protein processing/maturation
Sez_0577	345aa	32-33: YYI-EM	--	Lon-like protease
Sez_0608	441aa	31-32: ARA-EH	LPATG	SclZ.7, streptococcal collagen-like protein of zooepidemicus 7
Sez_0623	290aa	26-27: VTA-EQ	LPLTG	conserved protein function unknown
Sez_0625	280aa	25-26: VLA-VL	--	lipase
Sez_0668	387aa	27-28: FPA-SE	--	SdzD, streptodornase D of zooepidemicus
Sez_0696	445aa	46-47: VGA-LD	LPTTG	Zag, zooepidemicus albumin/IgG-binding protein
Sez_0709	282aa	28-29: AHS-DN	--	muramidase
Sez_0729	343aa	36-37: VQA-SD	LPSTG	SclZ.8, streptococcal collagen-like protein of zooepidemicus 8
Sez_0736	516aa	28-29: VLA-DS	--	Zn ABC transporter substrate-binding lipoprotein
Sez_0755	252aa	29-30: VDA-AR	--	SdzA, streptodornase A of zooepidemicus
Sez_0772	91aa	34-35: VFA-AT	--	hypothetical protein function unknown
Sez_0781	926aa	27-28: VTA-QS	LPKTG	extracellular nuclease
Sez_0809	377aa	33-34: VAA-DT	LPSTG	M-protein ortholog, Szp
Sez_0811	665aa	38-39: VRA-TN	LPTDG	FszA (Cnz), collagen-binding fimbrial subunit protein A of zooepidemicus
Sez_0812	478aa	25-26: AFA-DT	LPSTG	FszB, T-Ag-like fimbrial subunit protein B of zooepidemicus
Sez_0814	392aa	40-41: VSG-EV	--	IdeZ, IgG endopeptidase of zooepidemicus
Sez_0815	400aa	39-40: ISA-DS	LPSTG	hypothetical protein function unknown

Gene Tag	Length	Cleavage Site	CWA	Product/Function
Sez_0820	1238aa	49-50: AAA-ES	LPKTG	pullulanase
Sez_0826	713aa	21-22: YYC-CD	--	conserved protein function unknown
Sez_0861	225aa	28-29: VNA-NT	LPKTG	mucin-binding protein
*Sez_0872<br>Sez_0873	149aa<br>199aa	34-35: TYA-DD	--	IgG endopeptidase, Mac 1-like
Sez_0946	1123aa	37-38: VFA-EE	LPKTS	ScpZ, streptococcal C5a peptidase of zooepidemicus
Sez_0956	85aa	29-30: SSA-KK	--	hypothetical protein function unknown
Sez_0970	283aa	26-27: VFA-EN	FPKTG	bacteriocin immunity modification protein
Sez_1016	323aa	25-26: HAA-EN	--	conserved protein function unknown
Sez_1056	357aa	32-33: SSA-SQ	--	polyamine-binding protein
Sez_1069	268aa	31-32: SVA-DT	--	SdzB, streptodornase B of zooepidemicus
Sez_1073	1003aa	28-29: VFA-LE	LPKTG	RBC-binding protein
Sez_1088	668aa	27-28: VQA-DQ	LPATG	5'-nucleotidase
Sez_1102	199aa	36-37: ASA-KD	--	muramidase
Sez_1114	1161aa	32-33: VFA-EP	LPKTG	serine protease subtilisin-like
Sez_1122	420aa	41-42: TIS-DQ	--	conserved protein function unknown
Sez_1264	719aa	24-25: VFA-IE	--	alpha-amylase
Sez_1268	417aa	25-26: FSF-PT	--	DltD, D-Ala transfer protein
Sez_1299	1063aa	28-29: VLA-EE	--	hyaluronidase
Sez_1323	1048aa	27-28: YVA-TT	--	conserved protein function unknown
Sez_1356	134aa	32-33: SEA-VD	--	conserved protein function unknown
Sez_1363	108aa	26-27: VEA-FS	--	conserved protein function unknown
Sez_1385	101aa	20-21: VSA-AT	--	conserved protein function unknown
Sez_1421	498aa	31-32: VLA-EQ	--	SclZ.9, streptococcal collagen-like protein of zooepidemicus 9

Gene Tag	Length	Cleavage Site	CWA	Product/Function
Sez_1425	1546aa	42-43: VFA-ED	--	sialidase/neuraminidase
Sez_1457	666aa	31-32: VLA-EE	LPKTN	SclZ.10, streptococcal collagen-like protein of zooepidemicus 10
Sez_1480	238aa	22-23: VKA-DD	--	conserved protein function unknown
Sez_1481	200aa	28-29: STA-DQ	--	surface protein of unknown function, ErfK family
Sez_1582	275aa	29-30: VTA-RQ	--	ABC transporter AA-binding protein
Sez_1583	151aa	25-26: IYS-TD	--	hypothetical protein function unknown
Sez_1592	372aa	25-26: FVA-QK	--	conserved protein function unknown
*Sez_1598<br>Sez_1599	117aa<br>389aa	35-36: AHA-EE	LPATG	SclZ.11, streptococcal collagen-like protein of zooepidemicus 11
Sez_1698	428aa	38-39: AQA-DH	--	peptidoglycan D,D-carboxypeptidase
Sez_1699	411aa	21-22: SLA-ED	--	peptidoglycan D,D-carboxypeptidase
Sez_1700	393aa	23-24: VHA-ED	--	peptidoglycan D,D-carboxypeptidase
Sez_1710	522aa	24-25: VQA-DH	--	ABC transporter glutamine-binding protein
Sez_1724	469aa	38-39: ASA-QE	--	alpha-amylase
Sez_1735	803aa	20-21: TNA-AS	--	histidine triad extracellular protein
Sez_1737	515aa	28-29: YNT-YT	LPRSG	conserved protein function unknown
Sez_1748	52aa	35-36: SYA-NN	--	hypothetical protein function unknown
Sez_1752	265aa	29-30: VAA-QD	--	conserved protein function unknown
Sez_1759	482aa	35-36: AFA-EQ	LPKTH	fibronectin-binding protein, fimbrial subunit like
Sez_1775	424aa	36-37: VKA-NH	--	Skc, streptokinase plasminogen activator of GCS
Sez_1778	405aa	36-37: AEA-QL	LPSTG	streptococcal protective antigen-like surface protein
Sez_1802	457aa	42-43: AFA-DE	LPATG	surface protein M-like
Sez_1803	359aa	37-38: VKA-DQ	LPATG	SclZ.12, streptococcal collagen-like protein of zooepidemicus 12
Sez_1821	549aa	32-33: ILA-LS	LPSTG	FszC, fimbrial subunit protein C of zooepidemicus

Gene Tag	Length	Cleavage Site	CWA	Product/Function
Sez_1822	967aa	37-38: AFA-KD	LPSSG	FszD, fimbrial subunit protein D of zooepidemicus (collagen-binding?)
Sez_1825	832aa	30-31: LYA-AT	LPATG	fibronectin-binding protein
Sez_1828	304aa	27-28: IWA-QE	IPVTG	FszE, fimbrial subunit protein E of zooepidemicus (collagen-binding?)
Sez_1829	616aa	36-37: VKA-EE	IPNTG	FszF, fimbrial subunit protein F of zooepidemicus
Sez_1835	334aa	35-36: SKA-DS	--	conserved protein function unknown
Sez_1876	190aa	25-26: VLA-AD	--	SlaA, streptococcal phopholipase A2
Sez_1901	626aa	37-38: VYG-NV	LPATG	*S. equi* immunoreactive protein Se68.9 ortholog/ M-like
*Sez_1908<br>Sez_1909<br>Sez_1910	271aa<br>212aa<br>233aa	45-46: VYG-DV	LPATG	*S. equi* immunoreactive protein Se68.9 ortholog/ M-like
Sez_1936	203aa	31-32: AFS-SE	--	extracellular protein staphylococcal antigen A-like
Sez_1960	407aa	43-44: AKG-AT	--	serine protease processing/maturation of secreted proteins

* Unconfirmed fragmented gene in the sequence assembly.
