# Supplementary material for: Genome Sequence of a Lancefield Group C Streptococcus zooepidemicus Strain Causing Epidemic Nephritis: New Information about an Old Disease
Source: PLoS One. 2008 Aug 21;3(8):e3026. doi: 10.1371/journal.pone.0003026 (PMC2516327; doi:10.1371/journal.pone.0003026)
Supplement: Table S2 — Products with Predicted Lipidation Signal Sequence. Inferred lipoproteins with with canonical amino-terminal lipidation signal sequence (0.05 MB PDF) [file pone.0003026.s003.pdf]

Table S2. Products with Predicted Lipidation Signal Sequence

Gene Tag	Length	Cleavage Site	Function and/or Product
Sez_0195	416aa	30-31: LSA-CA	PG endopeptidase
Sez_0237	269aa	19-20: LVA-CG	membrane/secreted protein processing
Sez_0246	437aa	21-22: LAA-CG	ABC transporter NeuNAc-binding lipoprotein
Sez_0271	284aa	21-22: LAA-CG	5'-nucleotidase
Sez_0279	552aa	27-28: LMA-CG	ABC transporter oligopeptide-binding lipoprotein
Sez_0373	294aa	19-20: LSA-CV	ABC transporter Fe(3+)-binding lipoprotein
Sez_0462	291aa	19-20: LGA-CQ	conserved protein function unknown
Sez_0473	322aa	22-23: LGA-CG	ABC transporter D-ribose-binding lipoprotein
Sez_0503	361aa	25-26: LSA-CH	conserved protein function unknown
Sez_0559	223aa	26-27: LTA-CS	secreted protein processing/maturation
Sez_0666	333aa	22-23: LAA-CQ	secreted protein processing/maturation
Sez_0679	746aa	22-23: LTA-CQ	Internalin A-like protein
Sez_0813	269aa	15-16: VAG-CL	C-family fimbrial sortase
Sez_0831	270aa	21-22: LAA-CG	ABC transporter polar AA-binding lipoprotein
Sez_0888	278aa	21-22: LVA-CG	ABC transporter AA-binding lipoprotein
Sez_0911	288aa	22-23: LSA-CS	ABC transporter phosphate-binding lipoprotein
Sez_0936	350aa	20-21: LVG-CG	ABC transporter nucleoside-binding lipoprotein
Sez_1017	146aa	20-21: LSA-CD	hitidine triad lipoprotein
Sez_1018	313aa	25-26: LSA-CH	laminin-binding lipoprotein, Lmb
Sez_1097	236aa	18-19: LVA-CL	muramidase
Sez_1137	216aa	32-33: LSA-CS	conserved protein function unknown

Gene Tag	Length	Cleavage Site	Function and/or Product
Sez_1155	294aa	25-26: LAA-CA	ABC transporter cystine-binding lipoprotein
Sez_1257	417aa	23-24: LVG-CA	ABC transporter maltose/maltodextrin-binding lipoprotein
Sez_1266	419aa	24-25: LSA-CS	ABC transporter maltose/maltodextrin-binding lipoprotein
Sez_1367	290aa	21-22: LAA-CG	ABC transporter phosphate-binding lipoprotein
Sez_1456	540aa	19-20: LTA-CQ	ABC transporter oligopeptide-binding lipoprotein
Sez_1466	268aa	19-20: LSG-CE	cyclophilin-like cis-trans isomerase
Sez_1469	310aa	20-21: LVA-CS	ABC transporter Fe(3+)-binding lipoprotein
Sez_1501	496aa	37-38: LTA-CG	ABC transporter sugar-binding lipoprotein
Sez_1605	193aa	21-22: LSA-CR	conserved protein function unknown
Sez_1619	312aa	19-20: LVA-CS	ABC transporter Fe(3+)-binding lipoprotein
Sez_1654	306aa	23-24: LTG-CV	membrane/secreted protein processing
Sez_1679	281aa	21-22: LVA-CS	ABC transporter D-Met-binding lipoprotein
Sez_1680	281aa	22-23: LAA-CG	ABC transporter amino acid-binding lipoprotein
Sez_1697	658aa	22-23: LAA-CG	ABC transporter oligopeptide-binding lipoprotein
Sez_1736	313aa	26-27: LVA-CS	Zn/laminin-binding lipoprotein, LraI-like
Sez_1790	847aa	23-24: LTA-CG	Internalin A-like histidine triad lipoprotein
Sez_1838	320aa	19-20: LVA-CR	ABC transporter substrate-binding lipoprotein
Sez_1839	328aa	28-29: LTA-CR	ABC transporter substrate-binding lipoprotein
