# Supplementary material for: Genome Sequence of a Lancefield Group C Streptococcus zooepidemicus Strain Causing Epidemic Nephritis: New Information about an Old Disease
Source: PLoS One. 2008 Aug 21;3(8):e3026. doi: 10.1371/journal.pone.0003026 (PMC2516327; doi:10.1371/journal.pone.0003026)
Supplement: Table S3 — Products with Predicted Double-Glycine Secretion Signal Sequence. Inferred proteins with bacteriocin/competenece peptide-like double-glycine secretion signal sequence (0.04 MB PDF) [file pone.0003026.s004.pdf]

Table S3. Products with Predicted Double-Glycine Secretion Signal Sequence

Gene Tag	Length	Cleavage Site	Product/Function
Sez_0490	67aa	37-38: GG-CC	streptolysin S precursor, SagA
Sez_0518	46aa	19-20: GG-AW	putative competence stimulating peptide
Sez_0519	43aa	18-19: GG-FD	putative competence stimulating peptide
Sez_0520	55aa	18-19: GG-AV	putative competence stimulating peptide
Sez_0965	66aa	23-24: GG-WG	bacteriocin
Sez_0969	76aa	23-24: GG-NC	bacteriocin
Sez_1195	61aa	17-18: GG-TG	bacteriocin
Sez_1200	51aa	24-25: GG-WD	bacteriocin/phermone BlpC-like
Sez_1516	67aa	18-19: GG-NP	bacteriocin/phermone BlpN-like
Sez_1517	84aa	23-24: GG-KN	bacteriocin/phermone BlpM-like
Sez_1522	98aa	19-20: GG-KG	bacteriocin/phermone BlpC-like
Sez_1525	44aa	25-26: GG-WL	bacteriocin/phermone bovicin-like
Sez_1529	67aa	22-23: GG-KI	bacteriocin enhancer peptide ThmB-like
Sez_1530	85aa	24-25: GG-YS	bacteriocin ThmA-like
Sez_1606	64aa	12-13: GG-KN	bacteriocin/phermone BlpM-like
