# Supplementary material for: Genome Sequence of a Lancefield Group C Streptococcus zooepidemicus Strain Causing Epidemic Nephritis: New Information about an Old Disease
Source: PLoS One. 2008 Aug 21;3(8):e3026. doi: 10.1371/journal.pone.0003026 (PMC2516327; doi:10.1371/journal.pone.0003026)
Supplement: Table S4 — Insertion Sequence Elements (0.09 MB PDF) [file pone.0003026.s005.pdf]

Table S4. Insertion Sequence Elements

MGCS10565 Locus Tag	Length (aa)	ISFinder				Note	Intact or Fragmented
		Best Hit	Family	Group	Origin		
Sez_0095	186	IS1193D	ISL3		Streptococcus pneumoniae SP-VA96	IS1193D C-terminal fragment	IS1167/IS1193D-like fragments
Sez_0096	155	IS1193D	ISL3	-	Streptococcus thermophilus CNRZ368	IS1193D N-terminal fragment	IS1167/IS1193D-like fragments
Sez_0097	60	IS652	ISL3	-	Bacillus halodurans C-125	IS1193D-like fragment	IS1167/IS1193D-like fragments
Sez_0121	267	IS861B	IS3	IS150	Streptococcus agalactiae COH-I	IS861 orfB	intact
Sez_0122	171	IS861A	IS3	IS150	Streptococcus agalactiae COH-I	IS861 orfA	intact
Sez_0180	573	ISMbov3	IS1634	-	Mycoplasma bovis	IS4-like Desulfitobacterium hafniense	intact
Sez_0196	317	IS1239	IS30	-	Streptococcus pyogenes MGAS1898 (M15)	IS1239	intact
Sez_0210	476	IS663	ISNCY	-	Bacillus halodurans C-125	IS663-like	intact
Sez_0295	171	IS861A	IS3	IS150	Streptococcus agalactiae COH-I	IS861 orfA	intact
Sez_0296	267	IS861B	IS3	IS150	Streptococcus agalactiae COH-I	IS861 orfB	intact
Sez_0345	171	IS861A	IS3	IS150	Streptococcus agalactiae COH-I	IS861 orfA	intact
Sez_0346	267	IS861B	IS3	IS150	Streptococcus agalactiae COH-I	IS861 orfB	intact
Sez_0507	376	IS1548	ISAs1	-	Streptococcus agalactiae Mc1 serotype III	IS1548-like	intact
Sez_0529	430	IS1193D	ISL3	-	Streptococcus thermophilus CNRZ368	IS1193D	intact
Sez_0555	96	IS1167	ISL3	-	Streptococcus pneumoniae CP1200	IS1193D-like fragment	IS1167/IS1193D-like fragments
Sez_0556	78	ISSmu2	ISL3	-	Streptococcus mutans	IS1193D-like fragment	IS1167/IS1193D-like fragments
Sez_0557	231	IS1193D	ISL3	-	Streptococcus thermophilus CNRZ368	IS1193D-like fragment	IS1167/IS1193D-like fragments
Sez_0733	171	IS1A			Escherichia coli	IS1 orf A	intact
Sez_0734	239	IS1B			Escherichia coli	IS1 orf B	intact

MGCS10565 Locus Tag	Length (aa)	ISFinder				Note	Intact or Fragmented
		Best Hit	Family	Group	Origin		
Sez_0775	112	IS1193D	ISL3		Streptococcus thermophilus CNRZ368	IS1167/IS1193D-like fragment	IS1167/IS1193D-like fragments
Sez_0819	92	ISRhba1	IS1595		Rhodobacterales bacterium		fragment
Sez_0875	376	IS1548	ISAs1	-	Streptococcus agalactiae Mc1 serotype III	IS1548-like	intact
Sez_0955	317	IS1239	IS30	-	Streptococcus pyogenes MGAS1898 (M15)	IS1239	intact
Sez_0957	376	IS1548	ISAs1	-	Streptococcus agalactiae Mc1 serotype III	IS1548-like	intact
Sez_0980	573	ISMbov3	IS1634	-	Mycoplasma bovis	IS4-like Desulfitobacterium hafniense	intact
Sez_1096	137	ISBth5	IS4	IS231	Bacillus thuringiensis	IS4 family fragment	fragment
Sez_1112	267	IS861B	IS3	IS150	Streptococcus agalactiae COH-I	IS861 orfB	intact
Sez_1113	171	IS861A	IS3	IS150	Streptococcus agalactiae COH-I	IS861 orfA	intact
Sez_1115	317	IS1239	IS30	-	Streptococcus pyogenes MGAS1898 (M15)	IS1239	intact
Sez_1116	317	ISXc5	Tn3	ISXc4	Xanthomonas campestris pv. citri XW45 (pXW45J)		?
Sez_1141	232	IS1202	ISNCY	-	Streptococcus pneumoniae SSZ serotype 19F	IS1202 fragment	intact
Sez_1142	104	IS1202	ISNCY	-	Streptococcus pneumoniae SSZ serotype 19F	IS1202 fragment	intact
Sez_1143	150	IS1202	ISNCY	-	Streptococcus pneumoniae SSZ serotype 19F	IS1202 fragment	intact
Sez_1201	65	ISPlu5	IS200/IS605		Photorhabdus luminescens subsp. laumondii	IS200-like fragment	intact
Sez_1202	157	ISPlu5	IS200/IS605		Photorhabdus luminescens subsp. laumondii	IS200-like	intact
Sez_1325-1326						IG fragments	fragments
Sez_1342	267	IS861B	IS3	IS150	Streptococcus agalactiae COH-I	IS861 orfB	intact
Sez_1343	171	IS861A	IS3	IS150	Streptococcus agalactiae COH-I	IS861 orfA	intact
Sez_1348-1349	-		IS3			fragments	IG fragments

MGCS10565 Locus Tag	Length (aa)	ISFinder				Note	Intact or Fragmented
		Best Hit	Family	Group	Origin		
Sez_1362	573	ISMbov3	IS1634	-	Mycoplasma bovis	IS4-like Desulfitobacterium hafniense	intact
Sez_1458	71	IS1167	ISL3	-	Streptococcus pneumoniae CP1200	ISL3 family fragment	fragment
Sez_1461	317	IS1239	IS30	-	Streptococcus pyogenes MGAS1898 (M15)	IS1239	intact
Sez_1491	228	ISSau2-A	IS3	IS150	Staphylococcus aureus subsp. aureus	ISSth1 orfA	intact
Sez_1492	123	ISSau2-B	IS3	IS150	Staphylococcus aureus subsp. aureus	ISSth1 orfB	intact
Sez_1493	147	ISBcen10	IS3	IS150	Burkholderia cenocepacia	ISSth1 orfC	intact
Sez_1519	171	IS861A-like	IS3	IS150	Streptococcus agalactiae COH-I	IS861 orfA	intact
Sez_1520	278	IS861B-like	IS3	IS150	Streptococcus agalactiae COH-I	IS861 orfB	intact
Sez_1528	317	IS1239	IS30	-	Streptococcus pyogenes MGAS1898 (M15)	IS1239	intact
Sez_1533	171	IS861A	IS3	IS150	Streptococcus agalactiae COH-I	IS861 orfA	intact
Sez_1534	267	IS861B	IS3	IS150	Streptococcus agalactiae COH-I	IS861 orfB	intact
Sez_1544	122	ISSg1	ISL3	-	Streptococcus gordonii M5	ISL3 family fragment	fragment
Sez_1624-1625		IS1167	ISL3	-	Streptococcus pneumoniae CP1200	IG fragments	fragments
Sez_1652	573	ISMbov3	IS1634	-	Mycoplasma bovis	IS4-like Desulfitobacterium hafniense	intact
Sez_1738	376	IS1548	ISAs1	-	Streptococcus agalactiae Mc1 serotype III	IS1548-like	intact
Sez_1747	317	IS1239	IS30	-	Streptococcus pyogenes MGAS1898 (M15)	IS1239	intact
Sez_1840	317	IS1239	IS30	-	Streptococcus pyogenes MGAS1898 (M15)	IS1239	intact
Sez_1841	232	IS1202	ISNCY	-	Streptococcus pneumoniae SSZ serotype 19F	IS1202 fragment	intact
Sez_1842	104	IS1202	ISNCY	-	Streptococcus pneumoniae SSZ serotype 19F	IS1202 fragment	intact
Sez_1843	150	IS1202	ISNCY	-	Streptococcus pneumoniae SSZ serotype 19F	IS1202 fragment	intact

MGCS10565 Locus Tag	Length (aa)	ISFinder				Note	Intact or Fragmented
		Best Hit	Family	Group	Origin		
Sez_1868	147	ISBcen10	IS3	IS150	Burkholderia cenocepacia	ISSth1 orfC	intact
Sez_1869	123	ISSau2-B	IS3	IS150	Staphylococcus aureus subsp. aureus	ISSth1 orfB	intact
Sez_1870	228	ISSau2-A	IS3	IS150	Staphylococcus aureus subsp. aureus	ISSth1 orfA	intact
Sez_1871	171	IS861A	IS3	IS150	Streptococcus agalactiae COH-I	IS861 orfA	intact
Sez_1872	267	IS861B	IS3	IS150	Streptococcus agalactiae COH-I	IS861 orfB	intact
Sez_1877	317	IS1239	IS30	-	Streptococcus pyogenes MGAS1898 (M15)	IS1239	intact
Sez_1896	430	IS1193D	ISL3	-	Streptococcus thermophilus CNRZ368	IS1193D	intact
Sez_1923	430	IS1193D	ISL3	-	Streptococcus thermophilus CNRZ368	IS1193D	intact
