# Supplementary material for: Genome Sequence of a Lancefield Group C Streptococcus zooepidemicus Strain Causing Epidemic Nephritis: New Information about an Old Disease
Source: PLoS One. 2008 Aug 21;3(8):e3026. doi: 10.1371/journal.pone.0003026 (PMC2516327; doi:10.1371/journal.pone.0003026)
Supplement: Table S5 — Two component system regulator genes (0.09 MB DOC) [file pone.0003026.s006.doc]

**Table S5**. Two Component System Regulators

| **TCS** | **Homologous Product*** | **Gene Tag** | **Size (aa)** | **% ID** | **% SIM** | **Size (aa)** | **Gene Tag** | **Strain** | **Putative Processes Influenced** |
| --- | --- | --- | --- | --- | --- | --- | --- | --- | --- |
| 1 | FasB SK | Sez_0228 | 448 | 64 | 80 | 448 | Spy0219 | *S. pyogenes*  MGAS2096 | Fibronectin-binding proteins/pilin, streptokinase,  hemolytic activity expression in *S. pyogenes* |
|  | FasC SK | Sez_0229 | 432 | 61 | 79 | 427 | Spy0220 |  |  |
|  | FasA RR | Sez_0230 | 246 | 83 | 93 | 246 | Spy0221 |  |  |
| 2 | Unknown RR | Sez_0311 | 228 | 75 | 86 | 228 | gbs0429 | *S. agalactiae*  NEM316 | Unknown, has highest similarity with uncharacterized TCS of *S. agalactiae* |
|  | Unknown SK | Sez_0312 | 336 | 64 | 78 | 345 | gbs0430 |  |  |
| 3 | YesN RR | Sez_0561 | 278 | 82 | 92 | 246 | Spy1300 | *S. pyogenes*  MGAS2096 | Growth phase adaptation, carbohydrate utilization? |
|  | YesM SK | Sez_0562 | 572 | 78 | 87 | 577 | Spy1299 |  |  |
| 4 | Unknown SK | Sez_0582 | 370 | 41 | 60 | 361 | gbs0298 | *S. agalactiae*  NEM316 | Small peptide bacteriocin/competence/quorum sensing? |
|  | Unknown RR | Sez_0583 | 198 | 66 | 81 | 196 | gbs0299 |  |  |
| 5 | CiaR RR | Sez_0919 | 224 | 87 | 93 | 224 | Spy1007 | *S. pyogenes*  MGAS2096 | Cell envelope, early competence, penicillin susceptibility in *S. pyogenes* and/or *S. pneumoniae* |
|  | CiaH SK | Sez_0920 | 436 | 80 | 90 | 436 | Spy1006 |  |  |
| - | SilA-like orphan RR | Sez_0964 | 245 | 59 | 76 | 249 | *silA* | *S. pyogenes*  JS95 | Small peptide bacteriocin/competence/quorun sensing in streptococci |
| 6 | BceR RR | Sez_1004 | 221 | 69 | 82 | 222 | gbs0963 | *S. agalactiae*  NEM316 | Bacitracin transport in *S. agalactiae* and *S. mutans* |
|  | BceS SK | Sez_1005 | 325 | 48 | 68 | 312 | gbs0964 |  |  |
| 7 | YesN-like RR | Sez_1181 | 261 | 70 | 86 | 257 | gbs1934 | *S. agalactiae*  NEM316 | Unknown, has similarity with YesNM of *S. pyogenes* but lower than that of Sez_0561-62 |
|  | YesM-like SK | Sez_1182-  Sez_1183† | 96  452 | 63 | 79 | 549 | gbs1935 |  |  |
| 8 | PnpS/PhoR SK | Sez_1368 | 432 | 57 | 75 | 443 | SP_2083 | *S. pneumoniae*  TIGR4 | Phosphate uptake in *S. pneumoniae* |
|  | PnpR/PhoP RR | Sez_1369 | 247 | 81 | 91 | 235 | SP_2082 |  |  |
| 9 | VicK SK | Sez_1395 | 450 | 91 | 97 | 450 | Spy0455 | *S. pyogenes*  MGAS2096 | Osmotic stress response in *S. pyogenes* |
|  | VicR RR | Sez_1396 | 236 | 95 | 98 | 236 | Spy0454 |  |  |
| 10 | YesN-like RR | Sez_1497 | 494 | 76 | 86 | 495 | Spy1325 | *S. pyogenes*  MGAS2096 | Unknown, has similarity with YesNM of *S. pyogenes* but lower than that of Sez_0561-62 |
|  | YesM-like SK | Sez_1498 | 564 | 77 | 89 | 575 | Spy1326 |  |  |
| 11 | BlpH SK | Sez_1526 | 444 | 43 | 65 | 446 | SP_0527 | *S. pneumoniae*  TIGR4 | Small peptide bacteriocin/competence/quorun sensing in streptococci |
|  | BlpR RR | Sez_1527 | 249 | 56 | 73 | 245 | SP_0526 |  |  |
| 12 | YvqC RR | Sez_1553 | 197 | 92 | 97 | 215 | Spy1353 | *S. pyogenes*  MGAS2096 | Secretion stresses? |
|  | YvqE SK | Sez_1554 | 334 | 84 | 94 | 334 | Spy1354 |  |  |
| 13 | CovS SK | Sez_1665 | 496 | 84 | 92 | 500 | Spy0301 | *S. pyogenes*  MGAS2096 | Pleiotropic virulence factor regulation in *S. pyogenes* |
|  | CovR RR | Sez_1666 | 228 | 97 | 99 | 228 | Spy0300 |  |  |
| 14 | Unknown SK | Sez_1902 | 600 | 30 | 51 | 767 | BC0881 | *B. cereus* | Unkown, has the lowest similarity of all of these TCS to a TCS present in another species |
|  | Unknown RR | Sez_1903 | 199 | 32 | 55 | 209 | SAV1884 | *S. aureus* MU50 |  |

* Abreviations: SK - sensor kinase, RR - response regulator.

† Unconfirmed disrupted gene in the genome assembly.
