# Supplementary material for: Genome Sequence of a Lancefield Group C Streptococcus zooepidemicus Strain Causing Epidemic Nephritis: New Information about an Old Disease
Source: PLoS One. 2008 Aug 21;3(8):e3026. doi: 10.1371/journal.pone.0003026 (PMC2516327; doi:10.1371/journal.pone.0003026)
Supplement: Table S6 — Proven and putative virulence factor homologues (0.22 MB DOC) [file pone.0003026.s007.doc]

**Table S6. Proven and Putative Virulence Factor Homologues**

| **MGCS10565 Locus Tag** | **MGCS10565 Gene/Function** | **Export Motifs*** | **Length**  **(aa)** | **Hit Type*** | **% ID** | **MGAS2096 Locus Tag** | **MGAS2096 Gene/Function** | **Posited Virulence Function** |
| --- | --- | --- | --- | --- | --- | --- | --- | --- |
| Sez_0099 | SclZ.1, collagen-like protein 1 | SSP & CWA | 428 | NR | 28.1 | Spy0851 | SclB, collagen-like protein B | Adhesin |
| Sez_0100 | SclZ.2, collagen-like protein 2 | SSP & CWA | 383 | NR | 7.1 | Spy0851 | SclB, collagen-like protein B | Adhesin |
| Sez_0162 | SclZ.3, collagen-like protein 3 | SSP & CWA | 335 | NR | 8.8 | Spy0851 | SclB, collagen-like protein B | Adhesin |
| Sez_0163 | MgaZ, multi-gene activator-like |  | 505 | R | 43.2 | Spy1748* | Mga, multi-gene activator pleiotropic virulence regulator | Virulence regulation |
| Sez_0164 | SpaZ, streptococcal protective Ag-like | SSP & CWA | 392 | NR | 12.0 | Spy1748 | Emm, M protein | Adhesin, Resistance to immune response |
| Sez_0185 | Uncharacterized surface protein | SSP & CWA | 559 | NR | 20.8 | Spy1748 | Emm, M protein | Adhesin?, Zinc metalloprotease? |
| Sez_0190 | SclZ.4, collagen-like protein 4 | SSP & CWA | 462 | NR | 29.6 | Spy0851 | SclB, collagen-like protein B | Adhesin |
| Sez_0199 | HasA, HA capsule synthesis |  | 417 | R | 71.5 | Spy1882 | HasA, HA causule synthesis | Adhesin, Resistance to immune response |
| Sez_0200 | HasB, HA capsule synthesis |  | 401 | R | 63.2 | Spy1883 | HasB, HA capsule synthesis | Adhesin, Resistance to immune response |
| Sez_0201 | HasC, HA capsule synthesis |  | 300 | NR | 90.3 | Spy1884 | HacC, HA capsule synthesis | Adhesin, Resistance to immune response |
| Sez_0213 | Sclz.5, collagen-like protein 5 | SSP & CWA | 336 | NR | 14.6 | Spy0851 | SclB, collagen-like protein B | Adhesin |
| Sez_0265 | *S. uberis* adhesion molecule-like | SSP | 834 | R | 50.9 | Spy0247 | PrgA, surface exclusion protein | Adhesin, lactoferrin-binding protein |
| Sez_0270 | GAPDH/NAPlr, nephritis associated plamin(ogen) receptor | Extracellular | 336 | R | 85.7 | Spy0252 | GAPDH/NAPlr | Plasmin(ogen) binding protein, Dissemination |
| Sez_0313 | Fnz, fibronectin-binding protein | SSP & CWA | 576 | NR | 16.0 | Spy0110 | FCT, fibronectin-binding protein | Adhesin, fibronectin-binding protein |
| Sez_0324 | C3 degrading proteinase? |  | 251 | R | 69.7 | Spy1596 | Putative C3 degrading proteinase | Protease, Resistance to immune response |
| Sez_0338 | Emm-like protein | SSP & CWA | 326 | NR | 15.2 | Spy1748 | Emm, M protein | Adhesin, Resistance to immune response |
| Sez_0370 | Isp-like, immunogenic secreted protein | SSP | 471 | R | 61.7 | Spy1558 | Immunogenic secreted protein (not in Mga regulon) | Adhesin?, Amidase? |
| Sez_0371 | Shr, heme-binding receptor | SSP | 1251 | R | 58.5 | Spy1557 | Fe3+ siderophore | Iron acquisition |
| Sez_0394 | Sfs, fibronectin-binding protein | SSP | 335 | NR | 4.4 | Spy1639 | Hypothetical membrane spanning protein | Adhesin, Fibronectin-binding |
| Sez_0490 | SagA, streptolysin S (SLS) | GG | 68 | R | 32.5 | Spy0623 | SagA | Pore forming toxin streptolysin S precursor |
| Sez_0491 | SagB, SLS synthesis/processing /export |  | 316 | R | 73.1 | Spy0624 | SagB | SLS synthesis/processing /export |
| Sez_0492 | SagC, SLS synthesis/processing /export |  | 354 | R | 77.9 | Spy0625 | SagC | SLS synthesis/processing /export |
| Sez_0493 | SagD, SLS synthesis/processing /export |  | 452 | R | 87.2 | Spy0626 | SagD | SLS synthesis/processing /export |
| Sez_0494 | SagE, SLS synthesis/processing /export |  | 201 | R | 58.5 | Spy0627 | SagE | SLS synthesis/processing /export |
| Sez_0495 | SagG, SLS synthesis/processing /export |  | 228 | R | 50.2 | Spy0628 | SagG | SLS synthesis/processing /export |
| Sez_0496 | SagF, SLS synthesis/processing /export |  | 307 | R | 78.2 | Spy0629 | SagF | SLS synthesis/processing /export |
| Sez_0497 | SagH, SLS synthesis/processing /export |  | 375 | R | 75.3 | Spy0630 | SagH | SLS synthesis/processing /export |
| Sez_0498 | SagI, SLS synthesis/processing /export |  | 372 | R | 68.4 | Spy0631 | SagI | SLS synthesis/processing /export |
| Sez_0499 | Fnz-like, fibronectin-binding | Hic-like, factor H-binding inhibitor of complement | SSP & CWA | 482 | NR | 13.4 | Spy1710 | SclA, collagen-like protein A | Adhesin, Resistance to immune response |
| Sez_0500 | SclZ.6, collagen-like protein 6 with fibronectin-binding domain | SSP & CWA | 522 | R | 10.6 | Spy1710 | SclA, collagen-like protein A | Adhesin, Fibronectin-binding |
| Sez_0511 | ScpC/CepA/spyCEP | SSP & CWA | 1634 | R | 61.2 | Spy0361 | spyCEP/Interleukin 8 protease | Proteinase, Resistance to immune response |
| Sez_0513 | Albumin-binding protein? | SSP & CWA | 394 | NR | 21.0 | Spy1748 | Emm, M protein | Adhesin |
| Sez_0530 | EsxA, Esx family protein | ESAT_6 secreted | 98 | NR | 15.3 | Spy0241 | GTPase | Toxin? |
| Sez_0531 | EsaA, ESAT-6 system protein? |  | 1045 | NR | 3.2 | Spy1862 | Phage infection protein | Toxin synthesis/export? |
| Sez_0532 | ESAT-6 system protein? | SSP | 174 | NR | 7.7 | Spy0633 | Endo/Exonuclease | Toxin synthesis/export? |
| Sez_0533 | ESAT-6 system protein? |  | 92 | NR | 12.9 | Spy0361 | spyCEP/Interleukin 8 protease | Toxin? |
| Sez_0534 | EssB, ESAT-6 system protein? |  | 385 | NR | 3.8 | Spy1887 | Glucose uptake family protein | Toxin synthesis/export? |
| Sez_0535 | EssC, ESAT-6 system protein? |  | 1458 | NR | 8.9 | Spy0392 | FtsK, cell divivion protein | Toxin synthesis/export? |
| Sez_0536 | ESAT-6 system protein? |  | 109 | NR | 11.2 | Spy1577 | Hypothetical membrane spanning protein | Toxin? |
| Sez_0537 | EsxB, Esx family protein |  | 144 | NR | 14.3 | Spy0823 | ABC transporter ATP-binding protein | Toxin? |
| Sez_0538 | ESAT-6 system protein? |  | 94 | NR | 19.1 | Spy0247 | PrgA, surface exclusion protein | Toxin |
| Sez_0577 | Lon-like/SdrC-like protease | SSP | 345 | R | 79.4 | Spy1285 | Lon-like ATP-dependent endopeptidase | Proteinase, Dissemination |
| Sez_0608 | SclZ.7, collagen-like protein 7 | SSP & CWA | 441 | NR | 17.0 | Spy0851 | SclB, collagen-like protein B | Adhesin |
| Sez_0620 | HylA, hemolysin | GG | 275 | R | 76.0 | Spy1248 | Lantibiotic precursor | Pore forming toxin |
| Sez_0668 | Streptodornase, SdzD | SSP | 387 | R | 63.7 | Spy1441 | Streptodornase (Streptococcal deoxyribonuclease) | Dissemination, Resistance to immune response |
| Sez_0679 | Internalin A-like lipoprotein | LSP | 746 | R | 45.8 | Spy1171 | Internalin protein | Adhesin/Invasin |
| Sez_0696 | Zag (GRAB-like), Macroglobulin/Albumin/IgG-binding | SSP & CWA | 445 | NR | 7.4 | Spy0851 | SclB, collagen-like protein B | Adhesin |
| Sez_0729 | SclZ.8 | SSP & CWA | 343 | NR | 14.1 | Spy0851 | SclB, collagen-like protein B | Adhesin |
| Sez_0736 | ZnuA, zinc-binding lipoprotein | SSP | 516 | R | 86.1 | Spy0604 | zinc uptake system protein | Adhesin |
| Sez_0755 | Streptodornase, SdzA | SSP | 252 | R | 84.5 | Spy0602 | Spd, streptococcal phage DNase | Disemination, Resistance to immune response |
| Sez_0774 | Eno, enolase | Extracellular | 435 | R | 98.6 | Spy0618 | Enolase | Plasmin(ogen)-binding, Dissemination |
| Sez_0809 | Szp | SSP & CWA | 377 | NR | 20.2 | Spy1748 | Emm, M protein | Adhesin, Resistance to immune response |
| Sez_0811 | FszA (Cnz), fimbrial subunit protein | SSP & CWA | 665 | NR | 7.7 | Spy0119 | FCT, collagen-binding protein | Adhesin |
| Sez_0812 | FszB, fimbrial subunit protein | SSP & CWA | 478 | NR | 5.1 | Spy0119 | FCT, collagen-binding protein | Adhesin |
| Sez_0814 | IdeZ, Mac-like | SSP | 392 | NR | 23.8 | Spy0739 | IgG endopeptidase | Proteinase, Resistance to immune response |
| Sez_0815 | Emm-like? | SSP & CWA | 400 | NR | 16.4 | Spy1748 | Emm, M protein | Adhesin, Resistance to immune response |
| Sez_0873-72† | Mac-like | SSP | 348 | R | 61.8 | Spy0739 | Mac protein | Proteinase, Resistance to immune response |
| Sez_0946 | ScpZ, C5a peptidase | SSP & CWA | 1123 | R | 34.6 | Spy1744 | ScpA, C5a peptidase | Pore forming toxin |
| Sez_0959 | HylIII hemolysin | GG | 216 | R | 86.1 | Spy0954 | HylIII, hemolysin III-like | Proteinase, Resistance to immune response |
| Sez_1002 | BceA, bacitracin resitance |  | 250 | NR | 37.3 | Spy1755 | ABC transporter ATP-binding protein | Antibiotic resistance |
| Sez_1003 | BceB, bacitracin resitance |  | 651 | R | 14.9 | Spy1652 | SalY, salvaricin A ABC transporter permease protein | Antibiotic resistance |
| Sez_1004 | BceR, bacitracin resitance |  | 221 | NR | 31.9 | Spy0454 | VikR, TCS response regulator | Antibiotic resistance |
| Sez_1005 | BceS, bacitracin resitance |  | 325 | NR | 18.2 | Spy1107 | TCS sensor histidine kinase | Antibiotic resistance |
| Sez_1017 | Htp, histidine triad protein | LSP | 146 | NR | 16.1 | Spy1738 | Htp, histidine triad protein | Adhesin |
| Sez_1018 | Lmb, laminin-binging protein | LSP | 313 | NR | 51.8 | Spy1740 | Lmb, Laminin-binding surface protein | Adhesin |
| Sez_1069 | Streptodornase, SdzB | SSP | 268 | R | 34.4 | Spy1772 | Streptodornase (streptococcal deoxyribonuclease) | Dissemination, Resistance to immune response |
| Sez_1073 | RBC-binding protein | SSP & CWA | 1003 | R | 50.2 | Spy0719 | Cell surface protein | Adhesin |
| Sez_1114 | Internalin A-like protein | SSP & CWA | 1161 | NR | 11.5 | Spy1748 | Emm, M protein | Adhesin/Invasin |
| Sez_1299 | Hyaluronidase | SSP | 1063 | R | 24.8 | Spy0830 | Hyaluronate lyase precursor | Glycosidase, Dissemination |
| Sez_1323 | Fibronectin-binding protein? | SSP | 1048 | NR | 3.5 | Spy0472 | chromosome segregation ATPase | Adhesin |
| Sez_1352 | Peptidase/Collagenase | ? | 428 | R | 93.0 | Spy0502 | Peptidase family U32 | Proteinase, Dissemination |
| Sez_1353 | Peptidase/Collagenase | ? | 308 | R | 88.3 | Spy0501 | Peptidase family U32 | Proteinase, Dissemination |
| Sez_1421 | SclZ.9, collagen-like protein 9 | SSP & CWA | 498 | NR | 14.7 | Spy1710 | SclA, collagen-like protein A | Adhesin |
| Sez_1425 | Sialidase/Neuraminidase | SSP | 1546 | NR | 1.3 | Spy0488 | beta-glucodise PTS IIABC | Glycosidase, Resistance to immune response |
| Sez_1457 | SclZ.10, collagen-like protein 10 | SSP & CWA | 666 | NR | 11.5 | Spy0851 | SclB, collagen-like protein B | Adhesin |
| Sez_1467 | MtsC, metal transporter |  | 277 | R | 89.6 | Spy0390 | MtsC, Metal transporter of streptococcus protein | Dissemination, Resistance to immune response |
| Sez_1468 | MtsB, metal trasporter |  | 241 | R | 79.3 | Spy0389 | MtsB, Metal transporter of streptococcus protein | Dissemination, Resistance to immune response |
| Sez_1469 | MtsA, metal transport | LSP | 310 | R | 90.3 | Spy0388 | MtsA, Metal transporter of streptococcus protein | Dissemination, Resistance to immune response |
| Sez_1470 | MtsR, metal transport regulator |  | 215 | R | 48.5 | Spy0386 | MtsR, Metal transporter of streptococcus protein | Dissemination, Resistance to immune response |
| Sez_1478 | HylX, hemolysin | GG? | 232 | R | 72.4 | Spy0378 | Hemolysin | Pore forming toxin |
| Sez_1507 | Hyaluronidase | ? | 564 | R | 52.1 | Spy1334 | Hyaluronoglucosaminodase | Glycosidase, Dissemination |
| Sez_1598-99† | SclZ.11, collagen-like protein 11 | SSP & CWA | 506 | NR | 13.0 | Spy1710-Spy0851 | SclA, collagen-like protein A | Adhesin |
| Sez_1626 | HylX, hemolysin | ? | 444 | R | 89.6 | Spy0334 | Hemolysin | Pore forming toxin |
| Sez_1735 | Htp, histidine triad protein | SSP | 803 | R | 47.0 | Spy1738 | Htp, histidine triad protein | Adhesin |
| Sez_1736 | Lmb, laminin-binging protein | LSP | 313 | R | 76.2 | Spy1740 | Lmb, Laminin-binding protein | Adhesin |
| Sez_1737 | Cell surface adhesin | SSP & CWA | 515 | NR | 4.7 | Spy1604 | Transcriptional regulator | Adhesin |
| Sez_1759 | FBP | SSP & CWA | 482 | NR | 17.0 | Spy0110 | FCT, fibronectin-binding protein | Adhesin, |
| Sez_1775 | Skc/Skz, streptokinase | SSP | 424 | NR | 2.5 | Spy0400 | peptide Met sulfoxide reductase | Plasminogen proteinase activator, Dissemination |
| Sez_1778 | Spa/M-like | SSP & CWA | 405 | R | 27.1 | Spy1748 | Emm, M protein | Adhesin, Resistance to immune response |
| Sez_1790 | Internalin A-like protein | LSP | 847 | NR | 17.6 | Spy1171 | Internalin protein | Adhesin/Invasin |
| Sez_1802 | Emm-like? | SSP & CWA | 457 | NR | 23.1 | Spy1748 | Emm, M protein | Adhesin, Resistance to immune response |
| Sez_1803 | SclZ.12, collagen-like protein | SSP & CWA | 359 | NR | 14.1 | Spy1710 | SclA, collagen-like protein A | Adhesin, Resistance to immune response |
| Sez_1821 | FszC, fimbrial subunit protein | SSP & CWA | 549 | NR | 7.2 | Spy0119 | FCT, collagen-binding protein | Adhesin, Resistance to immune response |
| Sez_1822 | FszD, fimbrial subunit protein | SSP & CWA | 967 | NR | 2.0 | Spy0119 | FCT, collagen-binding protein | Adhesin, Resistance to immune response |
| Sez_1825 | Fironectin-bnding protein | SSP & CWA | 832 | NR | 17.2 | Spy0119 | FCT, collagen-binding protein | Adhesin, Resistance to immune response |
| Sez_1828 | FszE, fimbrial subunit protein | SSP & CWA | 304 | NR | 6.9 | Spy0119 | FCT, collagen-binding protein | Adhesin, Resistance to immune response |
| Sez_1829 | FszF, fimbrial subunit protein | SSP & CWA | 616 | NR | 3.2 | Spy1781 | Sorbitol operon regulator | Adhesin, Resistance to immune response |
| Sez_1876 | Sla, streptococcal phopholipase A_2_-like | SSP | 190 | NR | 12.1 | Spy0590 | Sla, phospholipase A_2_ | Toxin, Phospholipase adherence modulator |
| Sez_1901 | M-like | SSP & CWA | 626 | NR | 22.5 | Spy1748 | Emm, M protein | Adhesin, Resistance to immune response |
| Sez_1908-09-10† | M-like | SSP & CWA | 716 | NR | 23.5 | Spy1748 | Emm, M protein | Adhesin, Resistance to immune response |
| Sez_1960 | Serine protease HtrA | SSP | 407 | R | 74.2 | Spy1897 | HtrA serine protease | Proteinase, Dissemination |

* Abbreviations: SSP, secretion signal peptide; CWA, cell wall anchor; LSP, lipidation signal peptide; GG, double-gylcine leader peptide; R, reciprocal best hit; NR, non-reciprocal best hit; ?, expected extracellular but lacks canonical secretion signal sequences.

† Unconfirmed fragmented gene in the sequence assembly.
